# STAG2-truncating variants reveal a mosaic STAG2 inactivation pattern and compensatory mechanisms involving cohesin complex remodeling

**DOI:** 10.1016/j.isci.2025.114195

**Published:** 2025-11-22

**Authors:** Macarena Moronta Gines, Marja W. Wessels, Valentina Casa, Thomas van Staveren, Amber Hof, Wendy K. Chung, Marjolaine Willems, Anna Sandestig, Irina Huening, Peter Turnpenny, Mathilde Lefebvre, Ilaria Parenti, Frank J. Kaiser, Jeroen Demmers, Wilfred F.J. van Ijcken, Kerstin S. Wendt

**Affiliations:** 1 Department of Cell Biology, Erasmus MC, 3015 GD Rotterdam, the Netherlands; 2 Department of Developmental Biology, Erasmus MC, 3015 GD Rotterdam, the Netherlands; 3 Department of Clinical Genetics, Erasmus MC, 3015 GD Rotterdam, the Netherlands; 4 Department of Pediatrics, Boston Children’s Hospital, Harvard Medical School, 300 Longwood Avenue, Boston, MA 02115, USA; 5 Génétique Médicale, Reference Center for Constitutional Bone Diseases, CHU de MONTPELLIER, Institute for Neurosciences of Montpellier, University Montpellier, INSERM, 34295 Montpellier Cedex 05, France; 6 Clinical Department of Clinical Genetics, Region Östergötland, 581 85 Linköping, Sweden; 7 Institute of Human Genetics, University of Lübeck, 23562 Lübeck, Germany; 8 MVZ für Humangenetik und Molekularpathologie, 18059 Rostock, Germany; 9 Clinical Genetics, Royal Devon University Healthcare NHS Foundation Trust, Exeter EX1 2ED, UK; 10 UF de Foetopathologie, CHU d’Orleans, 45067 Orleans Cedex 2, France; 11 Institut für Humangenetik, Universitätsklinikum Essen, Universität Duisburg-Essen, 45147 Essen, Germany; 12 Department of Biochemistry, Erasmus University Medical Center, 3015 GD Rotterdam, the Netherlands; 13 Proteomics Center, Erasmus University Medical Center, 3015 GD Rotterdam, the Netherlands; 14 Center for Biomics, Erasmus University Medical Center, 3015 GD, Rotterdam, the Netherlands; 15 Department of Biomedical and Clinical Sciences, Linköping University, Linköping, Sweden

**Keywords:** Health sciences, Medicine, clinical genetics, Biological sciences, Biochemistry, Physiology

## Abstract

Cohesin plays a central role in three-dimensional genome organization and transcriptional regulation, with functional diversity determined by incorporation of distinct STAG subunits. Pathogenic variants in the X-linked *STAG2* gene cause a rare cohesinopathy with variable clinical manifestations. Molecular analyses of fibroblasts from females carrying germline *STAG2*-truncating variants revealed highly skewed X chromosome inactivation favoring the mutant allele, resulting in loss of STAG2 expression in most cases. STAG2-deficient cells displayed a proliferative advantage and transcriptional alterations without detectable defects in sister chromatid cohesion or DNA repair. Notably, compensatory upregulation of *STAG1* and ectopic expression of the germ cell-specific paralog *STAG3* were observed, leading to the formation of a previously unrecognized chimeric cohesin complex in somatic cells. These findings suggest that females with *STAG2*-truncating variants exhibit mosaicism for STAG2 expression and compensatory STAG3 incorporation, providing mechanistic insight into the phenotypic variability observed in *STAG2*-associated cohesinopathies.

## Highlights

• Females with germline *STAG2*-truncating variants show skewed X chromosome inactivation
• STAG2-negative cells seem to have a proliferative advantage over STAG2-positive cells
• STAG3 is expressed in 2 of 4 skin fibroblast samples and might substitute for STAG2

## Graphical abstract

**Figure:**
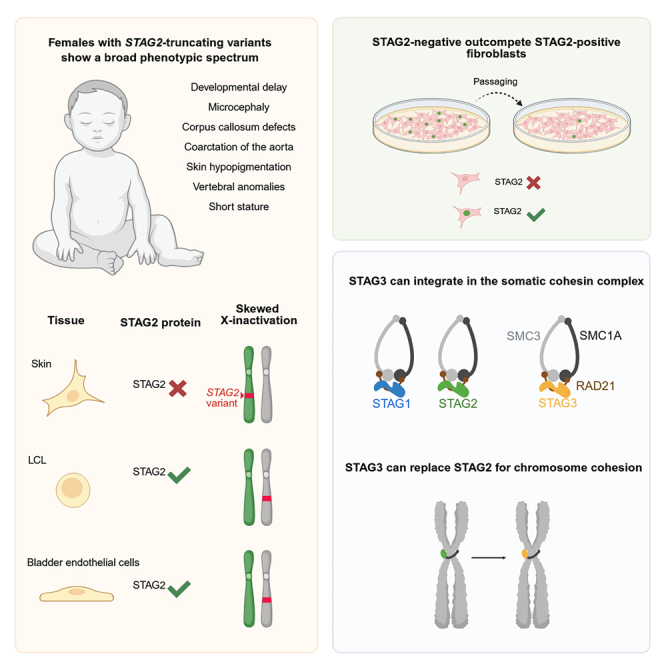

## Introduction

Cohesin is a multiprotein complex involved in various pathways to organize and protect the integrity of DNA in the cell nucleus. By tethering chromatin strands in *trans*, cohesin mediates sister chromatid cohesion to ensure correct chromosome segregation during cell division, and it facilitates DNA-damage repair. By connecting distal DNA segments on the same strand in a process termed loop extrusion, cohesin shapes the 3D organization of the chromatin fiber and impacts gene regulatory mechanisms.

Core subunits of the complex are SMC3, SMC1A, RAD21, and an SA/STAG (Stromal Antigen) subunit, while additional regulatory proteins (NIPBL, MAU2, HDAC8, ESCO1, ESCO2, WAPL, sororin) transiently associate to regulate cohesin’s association with chromatin (for a review see Hoencamp and Rowland^1^ and Davidson^2^).

Pathogenic variants in *NIPBL*, *MAU2*, *SMC1A*, *SMC3*, *RAD21,* and *HDAC8* cause Cornelia de Lange syndrome (CdLS), a multisystem disorder characterized by intellectual disability, facial dysmorphism, growth delay, and upper limb anomalies.^3^^,^^4^ Individuals with pathogenic variants in the STAG subunits display some overlapping features (growth defects and intellectual disability) but also some distinct ones (no facial phenotype or limb malformations).

Mammalian cells have three STAG paralogs—STAG1, STAG2, and STAG3. STAG1 and STAG2 are ubiquitously expressed in somatic cells, with varying ratios between cell types, and they are mutually exclusive integrated in the complex.^5^^,^^6^ STAG3 is found in germ cells and certain cancer cell lines,^7^^,^^8^ and only recently it was also observed in mouse embryonic stem cells.^9^ STAG1- and STAG2-containing cohesin complexes have distinct roles for sister chromatid cohesion, chromatin looping, and gene expression regulation. STAG1 mediates larger chromatin loops than STAG2, while chromatin interactions mediated by STAG2 often involve promoters and enhancers,^5^^,^^6^^,^^10^^,^^11^ suggesting a more prominent role for STAG2 in gene regulation. STAG2 is ubiquitously expressed, but expression levels vary between tissue types.^5^^,^^12^ STAG2 is also one of the most commonly mutated genes in various types of cancer.^13^^,^^14^ STAG3 is important for chromosome pairing and synapsis as well as DNA repair during meiosis.^15^

Germline pathogenic variants in *STAG1* have been reported in more than 20 affected individuals^16^^,^^17^^,^^18^^,^^19^^,^^20^ and are mainly associated with intellectual disability, in some cases also presenting with microcephaly or epilepsy.^17^ Germline *STAG3* pathogenic variants are associated with ovarian failure^21^ and spermatogenic failure.^22^^,^^23^^,^^24^ *STAG2* is located on Xq25 and undergoes X chromosome inactivation (XCI) in humans.^25^^,^^26^ Pathogenic variants in *STAG2* have been described in more than 25 patients to date.^27^^,^^28^^,^^29^^,^^30^^,^^31^^,^^32^^,^^33^^,^^34^ In the majority of cases, females present with truncating *STAG2* variants and males with missense substitutions. It is speculated that hemizygous truncating *STAG2* variants in males are either lethal or result in severe fetal outcomes, explaining why only male individuals harboring missense variants are reported.^28^ The diverse clinical spectrum of females with truncating *STAG2* variants includes developmental delay, intellectual disability, and congenital abnormalities including congenital heart defects, brain malformations, skeletal defects, and craniofacial features like holoprosencephaly.^34^

Here, we compared six previously unreported female individuals with germline *STAG2*-truncating variants and describe a hitherto unknown variation of the XCI pattern in affected females, leading to the full absence of STAG2 in skin fibroblasts. We find that STAG1 replaces STAG2 to some extent in these cells and also observe expression of the germ-cell specific STAG3, which seems to have a role in the fitness of those cells. The variable X-inactivation status between tissues and our observations concerning the proliferation capacity of STAG2-negative cells provide insight into the molecular mechanism underlying the broad and diverse phenotypic spectrum observed in the affected individuals.

## Results

### Clinical characterization of individuals diagnosed with *STAG2*-truncating variants

We identified six females with *de novo*-predicted truncating variants in *STAG2,* all of whom showed major congenital abnormalities. Clinical features in these individuals are summarized in Table 1 and below. Two of six presented with reduced fetal growth and were small for gestational age. Short stature was present in four of six patients (≤2 SDS, standard deviation score). Three of six had microcephaly (<2 SDS OFC, occipital frontal circumference more than 2 SDS below the mean for age and gender). All but one presented with developmental delay and intellectual disability; some exhibited autistic features and/or restless behavior. All individuals presented with congenital anomalies, affecting a wide range of organs. Congenital heart malformations, including coarctation of the aorta, ventricular septal defects (VSDs), and cardiomyopathy, were observed in two of six patients; hypomyelination and thin corpus callosum in three of six patients; vertebral anomalies in three out of six individuals; and hypopigmentation of the skin in two of six individuals. Diaphragmatic hernia, gut malrotation, coloboma of the optical nerve, renal cysts, and choanal atresia were each observed in one of six patients. Dysmorphic features were mild and diverse, including epicanthus, hypertelorism, blepharophimosis, and posteriorly rotated ears. These dysmorphic features were not suggestive of CdLS. All truncating *STAG2* variants are predicted to be damaging, and they are absent from gnomAD v.4.1.0.

**Table 1:** Phenotypes of individuals with heterozygous *STAG2* deletion or truncating variants

	Individual 1 (Pt1)	Individual 2 (Pt2)	Individual 3 (Pt3)	Individual 4 (Pt4)	Individual 5 (Pt5)	Individual 6 (Pt6)
Mutation type (NM_001042750.2)	160-kb deletion Xq25: del 5′ UTR and large part of the *STAG2* gene (chrX:122,899,890-123,059,602; NCBI36/hg18)	c.1840C>T; p.(Arg614∗)	c.646C>T; p.(Arg216∗)	c.3057T>A; p.(Tyr1019∗)	c.2857 C>T; p.(Arg953∗)	c.938del; p.(Gly313Alafs∗4)
Inheritance	*De novo*	*De novo*	Unknown (unaffected siblings)	*De novo*	*De novo*	*De novo*
Sex	Female	Female	Female	Female	Female fetus	Female
Age of patient	16 years	8 years	6 years	2 years	34 weeks’ gestation	10 years
X inactivation pattern^a^	Fibroblasts: nonrandom biopt1 96%/4%; biopt2 86%/14% (AR) Blood: nonrandom 99%/1% (AR) Urinary tract: random 64%/36% (AR)	Fibroblasts: nonrandom 80%/20% (AR)	Blood: nonrandom 96%/4% (AR) Fibroblasts: nonrandom 90%/10% (FMR1); 77%/32% (AR)	Blood: nonrandom 100%/0% (FMR1) fibroblasts: Nonrandom 71%/29% (FMR1)	Cord blood: nonrandom 92%/8% (AR) Liver: random 62%/38% (AR)	–
Pregnancy	Uneventful	IUGR, prenatal dx of diaphragmatic hernia	Uneventful	Uneventful Apgar 10/10	IUGR, prenatal dx of multiple malformations TOP at 34 weeks	Essentially uneventful but reduced fetal growth in 3^rd^ trimester
Birth weight	3,300 g at 40 weeks	–	3,100 g at term	3,100 g at 40 weeks	1,894 g at 34 weeks	3,020 g at 39 weeks
Growth						
Height	−3 SDS	−1 SDS	−1 to −2 SDS	−2 SDS	–	-2.9 SDS
Weight	0 SDS	−1.6 SDS	−1 SDS	−1.5 SDS	–	−2.4 SDS
OFC	0 SDS	−3 SDS	−2.5 SDS	−3.5 SDS	–	
Short stature	Yes	No	No	Yes	–	Yes
Microcephaly	No	Yes	Yes	Yes	–	No
Developmental delay	ID, IQ 62 no motor delay	IQ 79	Delayed speech HP:0000750 first words 18 months Motor delay HP:0001270 Autistic features HP:0000729 Restless behavior HP:0000752	No ID or motor delay, hyperkinesia HP:0002487	–	Delayed motor development HP:0001270 Delayed speech-language development HP:0000750 Walked at 21 months, overall mild developmental delay
Craniofacial anomalies	Ptosis R HP:0000508	Mild upslanting palpebral fissures HP:0000582	Temporal narrowing HP:0000341, Epicanthus HP:0000286, high palate HP:0000218, markedly posteriorly rotated ears HP:0000358, overfolded helix HP:0000396	Mild hypertelorism HP:0000316	Blepharophimosis HP:0000581, hypertelorism HP:0000316, epicanthus HP:0000286	Bilateral choanal atresia HP:0004502, Ankyloglossia HP:0010296, depressed nasal bridge HP:0005280, tubular nose, hypoplastic nasal alae HP:0000430, lateral extension of eyebrows HP:0011230
Limb malformation	Syndactyly digits 3 and 4 HP:0011939	–	No	–	–	–
Feeding problems	No	–	Unknown	Failure to thrive HP:0001508 Hypercaloric diet	–	At 2 years 4 months: nasal regurgitation HP:0011469, frequent choking and gagging HP:0030842, difficulties with solid food HP:0011968
Hearing impairment	Cochlear hearing loss left: 35 dB HP:0000407	–	No	–	–	Bilateral conductive hearing loss HP:0008513
Visual impairment	Hypermetropia, CHRPE HP:0000540	–	No	–	–	Hypermetropia HP:0000540
Skeletal abnormalities	–	Mild segment anomaly at T11, scoliosis HP:0002650	Not known, no radiology	Vertebral dysplasia HP:0003468, scoliosis HP:0002650	Hemivertebra T12 HP:0002937	–
Congenital heart disease	Coarctation of the aorta HP:0001680 Multiple muscular VSDs HP:0011625 Hypertrophic cardiomyopathy HP:0001639	No	No	Normal ultrasound	Coarctation of the aorta HP:0001680	Normal chocardiogram
Renal abnormalities	–	–	–	Cortical and medullary microcysts in both kidneys HP:0004734, HP:0008659	–	Normal renal scan
Brain (MRI) abnormalities	–	–	Generalized hypomyelination of subcortical white matter HP:0006808, Hypoplasia of corpus callosum HP:0002079	Thin corpus callosum HP:0033725, delayed myelination HP:0012448	Hypoplasia corpus callosum HP:0002079, Absence of the falx cerebri HP:0010654 Asymmetrical brain volume, abnormal brain maturation, possible stenosis of the right sinus piriformis HP:0025011, interhemispheric cyst HP:0032327	–
Abdominal abnormalities	–	Diaphragmatic hernia HP:0000776	–	–	Gut malrotation HP:0005211, liver cyst HP:0001407, polysplenia HP:0001748	–
Skin/hair abnormalities	–	–	Skin hypopigmentation Blaschko’s lines all over the body HP:0025293, HP:0001010, hair dyschromia	Hypopigmented skin patches HP:0001010	–	Thin nails HP:0001816 Thin hair HP:0002213
Other abnormalities	Delayed puberty HP:0000823	–	–	–	Coloboma opticus nerve	Sleeping difficulties, on melatonin in early childhood

CHRPE, congenital hypertrophy of retinal pigment epithelium; IUGR, intrauterine growth retardation; dx, diagnosis; SDS, standard deviation score; OFC, occipital frontal circumference; ID, intellectual disability; MRI, magnetic resonance imaging; Apgar, score to assess newborns based on appearance, pulse, grimace,activity and respiration.

a If the information is available, we report on the locus used for the X-inactivation analysis: AR, androgen receptor; FMR1, fragile X messenger ribonucleoprotein 1; biopt, skin biopsy.

**Figure 1:**
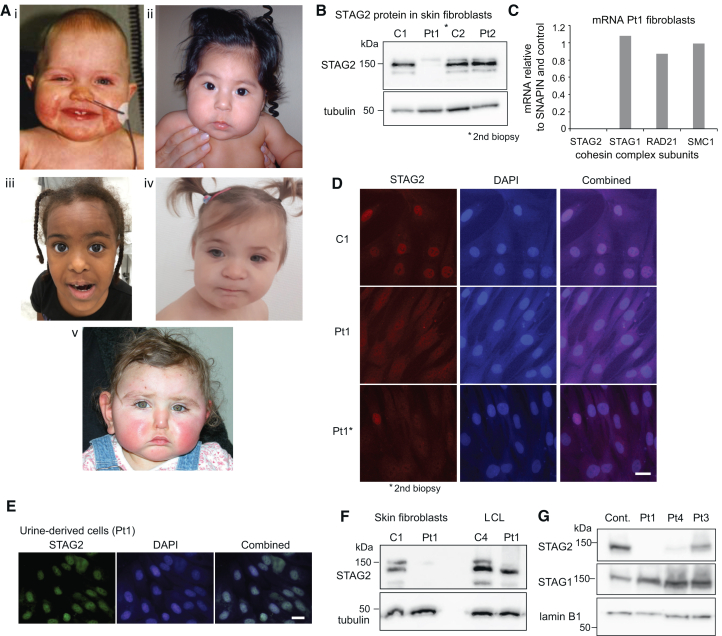
Facial phenotypes of STAG2-truncating variants and STAG2 expression in tissues (A) Childhood photographs of five individuals with truncating *STAG2* variants. Dysmorphic features included right ptosis at the age of 1 year (Pt1, i); upward slanting palpebral fissures at the age of 7 months (Pt2, ii); long face, temporal narrowing, and epicanthus at the age of 6 years (Pt3, iii); mild hypertelorism at the age of 3 years (Pt4, iv); and depressed nasal bridge, hypoplastic nasal alae, and lateral extension of eyebrows (Pt6, v). (B) Total cell extracts of two control fibroblasts lines (C1, C2) and patients (Pt1 and Pt2) were blotted for STAG2 and tubulin. (C) STAG2, STAG1, RAD21, and SMC1 mRNA levels were analyzed by RT-PCR/qPCR in Pt1 fibroblasts relative to the housekeeping gene SNAPIN and control fibroblasts (C1). (D) Immunostaining for STAG2 (red, Alexa594) in control fibroblasts (C1) and fibroblasts from two different biopsies from Pt1. Scale bar, 50 μm. (E) Immunostaining of STAG2 in cells grown from urine of Pt1. (F) Western blot comparing STAG2 protein levels in skin fibroblasts and blood-derived lymphoblastoid cells (LCL) from Pt1 with controls (C1 and C4). Tubulin is shown as loading control. Scale bar, 50 μm. (G) Western blots of total protein extracts of control (C1) and Pt1, Pt3, and Pt4 probed for STAG1 and STAG2. Laminin B1 is shown as loading control. See also Figures S1–S3.

Individual 1 (Pt1): This girl was diagnosed at 12 years of age with a *de novo* heterozygous 160-kb deletion in Xq25 (chrX:122,899,890-123,059,602; NCBI36/hg18), affecting the 5′ UTR, most of the *STAG2* gene and the *STAG2-AS1* gene (Figure S1A). This deletion is absent in the Database of Genomic Variants (DGV gold). She presented with mild dysmorphic features (Figure 1Ai; more details on the phenotype are given in the supplemental information and Table 1). In her first month she showed feeding problems and poor growth. Her echocardiogram showed coarctation of the aorta, patent ductus arteriosus, small muscular VSDs and hypertrophic cardiomyopathy. Coarctectomy and closure of the patent ductus were performed at the age of six weeks.

Individual 2 (Pt2): The girl had a *de novo* truncating *STAG2* variant (c.1840C>T; p.(Arg614∗)) that presumably leads to loss of function (LoF) (Figure 1Aii). She presented with congenital diaphragmatic hernia on fetal ultrasound. She was growth restricted throughout the pregnancy. Postnatally her diaphragmatic hernia was successfully surgically repaired. She was also found to have scoliosis with a mild vertebral anomaly at T11. She demonstrated mild developmental delay, and formal intelligence quotient (IQ) testing showed a full-scale IQ of 79. Her anthropometrics (height, weight, and head circumference) were consistently below average (for more details on the phenotype see Table 1).

Individual 3 (Pt3): This girl was diagnosed with a *STAG2* heterozygous nonsense variant (c.646C>T; p.(Arg216∗)) (Figure 1Aiii). She showed growth delay and delayed developmental milestones, intellectual disability, autistic features, restless behavior, postnatally developed microcephaly, and skin hypopigmentation in a pattern following Blaschko’s lines (Figure S1B) (more details on the phenotype in the supplemental information and Table 1).

Individual 4 (Pt4): This girl was diagnosed at 2 years of age with a *de novo STAG2* nonsense variant (c.3057T>A; p.(Tyr1019∗)) (Figure 1Aiv). She showed growth delay but no motor delay or intellectual disability and presented with vertebral dysplasia, scoliosis, and skin hypopigmentation (Figure S1C). The brain magnetic resonance imaging (MRI) revealed a thin corpus callosum and delayed myelination (more details on the phenotype in Table 1).

Individual 5 (Pt5): The patient was a female fetus at 34 weeks’ gestation diagnosed with a *de novo STAG2* nonsense variant (c.2857 C>T; p.(Arg953∗)). The fetus showed multiple malformations. Craniofacial abnormalities were blepharophimosis, hypertelorism, and epicanthus. Brain malformations included hypoplasia of the corpus callosum, absence of the falx cerebri, asymmetrical brain volume, abnormal brain maturation, and possible stenosis of the right sinus piriformis. Further, coarctation of the aorta, gut malrotation, liver cyst, and polysplenia were observed (more details on the phenotype in Table 1).

Individual 6 (Pt 6): The patient was a girl with a *de novo STAG2* frameshift variant (c.938del; p.(Gly313Alafs∗4)) born after an uneventful pregnancy, however with reduced fetal growth in the 3rd trimester. She demonstrated microcephaly and mild developmental delay with a delayed motor and speech development (Figure 1Av) (more details on the phenotype in Table 1).

### Loss of STAG2 expression in skin fibroblasts

The analysis of the STAG2 protein levels in the fibroblasts of Pt1 and Pt2 by western blotting unexpectedly revealed that the STAG2 protein was completely absent in Pt1 cells but was normal in early passages of Pt2 (Figure 1B, see also S2B). STAG2 mRNA was also absent in Pt1, while the mRNA levels of other cohesin complex components (STAG1, RAD21, and SMC1A) were comparable to the levels in control cells (Figures 1C and S4). The absence of STAG2-positive fibroblasts was observed in two independent skin biopsies taken from the inner left and right arm (Figure 1D). However, in endothelial cells grown from urine (Figure 1E) and blood-derived lymphoblastoid cells (LCLs) (Figure 1F) of Pt1, we observed the presence of STAG2.

We could obtain fibroblasts from two additional patients (Pt3 and Pt4) and observed reduced STAG2 protein levels also here, more pronounced in Pt4 but clearly visible in Pt3 (Figures 1G and S2A).

**Figure 2:**
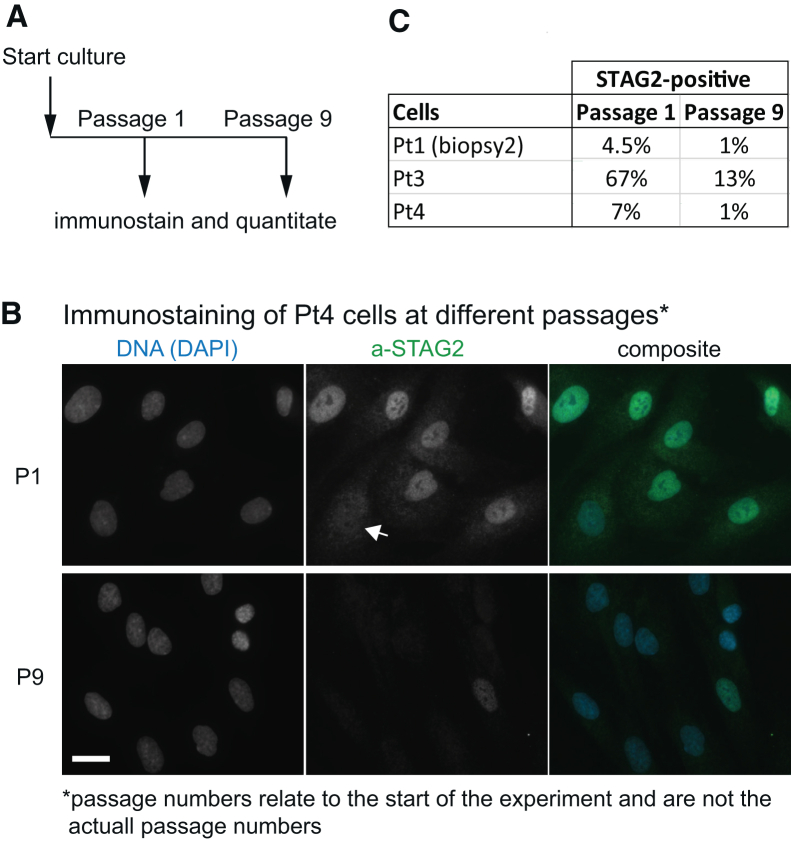
STAG2-positive cells are lost during prolonged culturing of patient cells (A) Scheme of the experimental setup. Note that passage numbers relate to the start of the experiment and are not the actual passage numbers. (B) Example of immunostaining for Pt4 cells. The arrow indicates a STAG2-negative cell. Scale bar, 50 μm. (C) Counts of the STAG2-positive cells for Pt1, Pt3, and Pt4 at passages 1 and 9.

In summary, three of four samples showed reduced STAG2 levels. Quantitation of the STAG2-positive cells observed by immunostaining (Figure 2) showed in early passages of the fibroblasts of Pt3 67% STAG2-positive cells versus 7% in Pt4 cells. For Pt1 we hardly observe STAG2-positive cells in the first biopsy (biopsy 1) but in the second biopsy 4.5% STAG2-positive cells were present (see also Figure 1D).

### Skewing of X-inactivation

Since *STAG2* is an X-linked gene and undergoes X inactivation, we analyzed the X-inactivation status in our samples. In blood of all analyzed cases, we observed a nearly full skewing of X inactivation (Table 1), presumably inactivating the defective allele since we observed normal STAG2 levels in LCLs from Pt1 (Figure 1F). However, for Pt1 we also observed a nearly complete skewing in fibroblasts (96/4 in biopsy 1), but this time presumably the intact allele was inactivated since STAG2 expression lacked in these cells (Figure 1F). For Pt2, which showed normal STAG2 expression levels by western blotting (Figure 1B), a ratio of 80/20 was observed and inactivation of the defective allele is presumed. Fibroblasts of Pt3 and Pt4 show skewing rates of 90/10 and 71/29, respectively, again presumably inactivating the intact allele since loss of STAG2 expression is observed (Figure 1G). Note that the X-inactivation status for Pt3 was determined at a later passage than for the other fibroblasts. In summary, for all analyzed cases we observe a non-normal skewing of X inactivation, in opposite directions between blood and skin fibroblasts, although we could only demonstrate this for Pt1.

### STAG2 deficiency might present a proliferative advantage

To understand how STAG2-deficient cells can become dominant in a cell population, we wanted to assess the proliferation of these cells. The presence of STAG2-positive and STAG2-negative fibroblasts in cultures of Pt1, Pt3 (Figures 1D and S2A), and Pt4 (Figure S2A) gives us the unique opportunity to assess isogenic STAG2-positive and STAG2-negative cells with identical history concerning passage number and culture conditions. This is important since proliferation of fibroblast cultures depends on many factors, including donor age, genetic background, and passage number.

To test whether STAG2- positive and -negative cells proliferate similarly, we cultured Pt1 fibroblasts over several passages (Figure 2A) and quantitated the number of STAG2-positive cells by immunostaining (Figure 2B). The percentage of STAG2-positive fibroblasts decreased from about 4.5% to 1% within nine passages for Pt1; for Pt4 we observed a decrease from 7% to 1% STAG2-positive cells. For Pt3, STAG2-positive cells were dominating in early passages, but we observed a drop from 67% to 13% after nine passages (Figure 2C). The consistent observations from these three fibroblast samples suggested a proliferative advantage of STAG2-negative fibroblasts, at least under culture conditions. During the revision of this manuscript, we discovered that the culture of Pt2 also acquired STAG2-negative cells (only 72% STAG2-positive cells at passage 6). In all control fibroblasts of this study, we did not observe STAG2-negative cells, even at high passage numbers (>20). For C1 and C2, we analyzed >100 cells from independent cultures.

### STAG2 deficiency does not lead to delayed DNA-damage repair

Since STAG2 was shown to be involved in DNA-damage repair^35^^,^^36^ and important for sister chromatid cohesion,^37^ we tested the cells for those functions. The DNA-damage repair capability was tested by quantitating the γH2AX signal after induction of DNA damage with gamma irradiation in fibroblasts of Pt1, Pt2, and Pt3 plus two control fibroblast lines (C1 and C2). In all tested cells the γH2AX foci induced by gamma irradiation disappeared with a similar kinetics and dropped to the level before irradiation within 8 h (Figure S3A). Spreads of mitotic chromosomes prepared from patients’ fibroblasts and controls did not show signs of centromere defects or other indications of premature loss of sister chromatid cohesion (Figure S3B).

### STAG2 is partially replaced by STAG1 at genomic-binding sites

**Figure 3:**
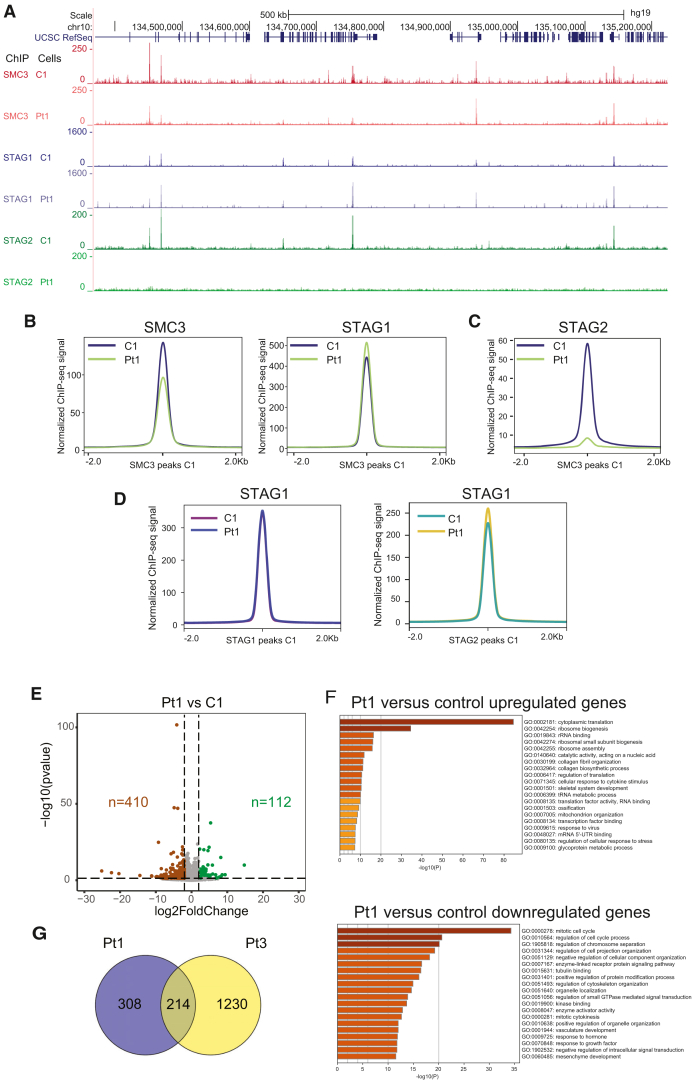
Fibroblasts of Pt1 show weakened cohesin binding and altered gene expression (A) ChIP-seq for SMC3, STAG1, and STAG2 was performed from C1 (control) and Pt1 fibroblasts. (B) Accumulated peak signals for SMC3 and STAG1 plotted on SMC3 peaks present in C1 and Pt1. (C) Accumulated peak signals for STAG2 plotted on SMC3 peaks. (D) STAG1 signal in C1 and Pt1 plotted over STAG1 and STAG2 peaks called in C1. (E) Volcano plot of differentially expressed genes between Pt1 and controls. Significant genes with FC>|2| and *p* value <0.05 are colorized. (F) GO terms enriched for up- and downregulated genes obtained with Metascape. (G) Venn diagram showing the overlap of misregulated genes between Pt1 and Pt3. The gene set overlap test (hypergeometric intersection) showed a significance of *p* < 1 × 10^−16^ for the overlapping genes. See also Figures S4–S6.

Previously, we and others have shown that STAG1-cohesin and STAG2-cohesin localize to the same sites but localize there exclusively. STAG1 can to some extent replace STAG2, but not vice versa*.*^6^ To test the occupancy of cohesin sites in Pt1 fibroblasts, we performed chromatin immunoprecipitation (ChIP) sequencing (ChIP-seq) for SMC3, STAG2, and STAG1 in Pt1 and control fibroblasts (Figure 3A). As expected, we do not observe STAG2 peaks in the Pt1 sample (Figure 3A). Since we cannot exclude that variation in binding sites can be caused by different genetic backgrounds, we analyzed only SMC3 peaks (20,914 peaks) and STAG1 peaks (14,967 peaks) that are shared between C1 and Pt1.

The aggregate peak analysis shows somewhat lower SMC3 occupancy on SMC3 peaks in Pt1 than for C1 (Figure 3B), a slightly increased STAG1 occupancy on SMC3 peaks in Pt1, and no signal for STAG2 in Pt1 (Figure 3C). The STAG1 occupancy on STAG1 peaks does not change between C1 and Pt1 (Figure 3D), while we see an increased STAG1 occupancy on the STAG2 peaks observed in C1. We further noted that most binding sites are co-occupied by SMC3, STAG1, and STAG2 in the control, as observed for other human cell lines in multiple other studies (e.g., Casa et al.^6^). Together, the reduction in SMC3 occupancy and the increase in STAG1 indicate that STAG1 takes STAG2 sites, although not completely. It is important to note that the mRNA levels for SMC3 and STAG1 are unchanged in Pt1 (Figure S4), so there is no compensatory regulation of those genes in the absence of STAG2.

### Changes in gene expression patterns are consistent with altered cell proliferation

To investigate whether the different degrees of STAG2 deficiency observed in the fibroblasts of Pt1 and Pt3 are linked to a transcriptome change, we performed RNA sequencing for these lines and for four female control fibroblast lines. For Pt1, fibroblasts from both biopsies were sequenced. All lines were cultured in parallel to identical confluency before preparing the RNA.

With a stringent cutoff of log2 fold change >2 and *p* value <0.05, we observed for Pt1 112 upregulated and 410 downregulated genes (Figure 3E). Interestingly, Gene Ontology (GO) terms associated with downregulated genes were linked to cell cycle regulation, and upregulated genes were linked to ribosome biogenesis and translation (Figure 3F). Pt4 shows the same loss of STAG2 expression as Pt1, but was received only after completion of the RNA sequencing. To obtain an indication whether the transcriptome of those cells is similar to Pt1, five downregulated genes with STAG2 sites near the promoter in control cells (C1) (Figure S5A) were tested by reverse-transcription PCR (RT-PCR)/qPCR. Selected genes were *PRDM8*, encoding a histone methyltransferase involved in neuronal development^38^; *NCEH1*, encoding a cholesterol ester hydrolase involved in the cholesterol metabolism^39^; *RNF182*, encoding an E3 Ubiquitin-Protein Ligase involved in RETT syndrome^40^; *GREB1L*, encoding the GREB1-like Retinoic Acid Receptor Coactivator and associated with different congenital conditions^41^; and *CARMIL1* (encoding Capping Protein Regulator And Myosin 1 Linker 1), a gene important for cell morphology.^42^ All genes are downregulated in Pt4 to a similar extent as in Pt1 (Figure S5B).

For Pt3 fibroblast (STAG2 protein absent in some cells), we observed 708 downregulated and 736 upregulated genes that are linked to diverse GO terms (Figure S6A). Between Pt1 and Pt3, we observed 214 common misregulated genes (Figure 3G), with 20 common upregulated genes and 130 common downregulated genes (Figure S6B).

### Germ-cell-specific STAG3 is expressed in skin fibroblasts

To assess the composition of the cohesin complex in the absence of STAG2, we immunoprecipitated SMC3 from lysates of Pt1 as well as control fibroblasts and identified copurifying STAG proteins by mass spectrometry (Table S2). We observed an increased abundance of STAG1 and, surprisingly, also identified the germ-cell specific STAG paralog STAG3. Recently, STAG3 expression has been reported for cancer cells^43^ but is unknown for healthy somatic cells.

**Figure 4:**
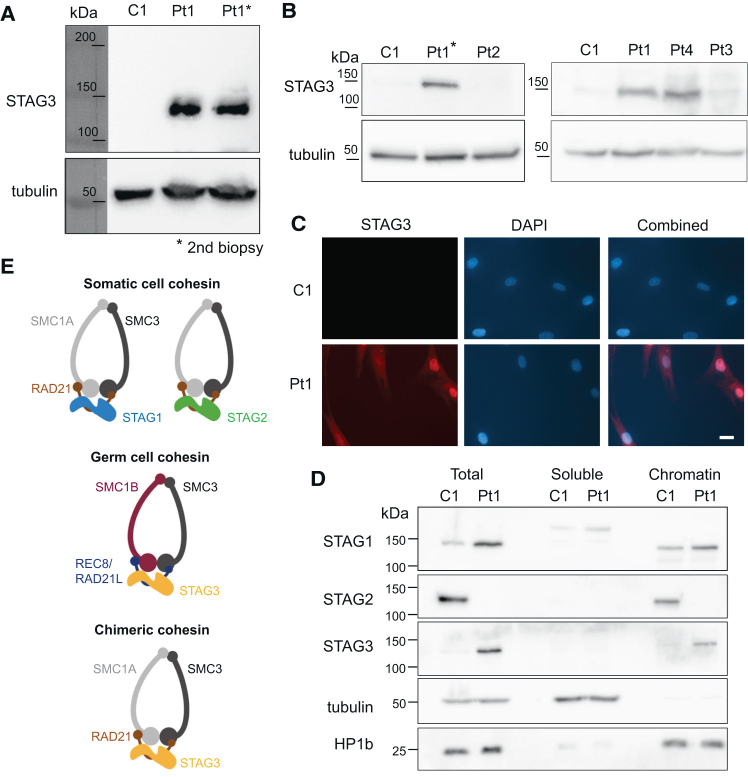
Expression of germ-cell-specific STAG3 in STAG2-deficient fibroblasts (A) Western blot detects STAG3 in both skin biopsies of Pt1 but not in the control. Tubulin is shown as loading control. (B) STAG3 can be detected in Pt1 and Pt4 but not in Pt2 and Pt3 fibroblasts. (C) Immunostaining for STAG3 of control and Pt1 fibroblasts. Scale bar, 50 μm. (D) Fractionation of Pt1 and control (C1) fibroblasts in soluble and insoluble fraction indicated chromatin binding of STAG3. Tubulin is shown as loading control for the soluble fraction and HP1b for the insoluble (chromatin) fraction. (E) Models of cohesin complexes observed in somatic cells and germ cells, and the resulting chimeric somatic cell complex integrating STAG3.

Western blotting of total protein extracts showed that STAG3 is present in biopsies of both Pt1 (Figure 4A) and Pt4 but is absent from Pt2 and Pt3 and from controls (Figure 4B). We confirmed this by immunostaining for Pt1 (Figure 4C). Fractionation of the fibroblasts into soluble and chromatin-bound proteins shows that STAG3 associates with chromatin in Pt1 fibroblasts, together with the expected increased level of STAG1 (Figure 4D). We could not detect other germ-cell-specific cohesin components (SMC1B, REC8, and RAD21L) by western blotting (not shown) or in the mass spectrometry analyses that led to the observation of STAG3 (Tables S2, S3, and S4). STAG3 might, therefore, integrate and replace STAG2 in the cohesin complex and form a chimeric complex consisting of somatic and germ-cell-specific components (Figure 4E).

We tested multiple approaches to identify STAG3-binding sites in those cells (ChIP-seq, Cut&Run) but failed, most likely due to the limited performance of the available anti-STAG3 antibodies.

### STAG3 can not only rescue mitotic STAG2 function but also can cause proliferation defects

**Figure 5:**
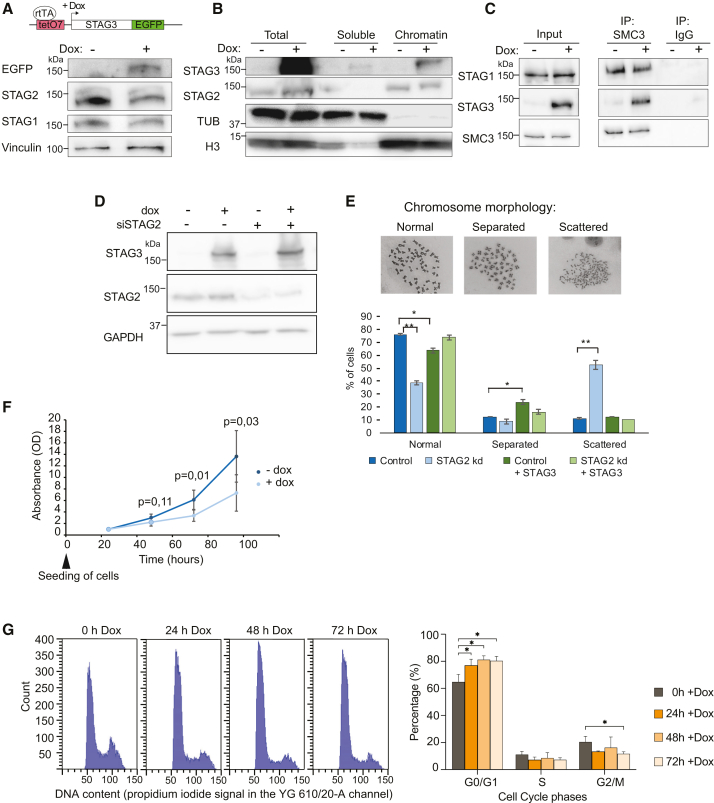
Effects of STAG3 overexpression in somatic cells (A) Scheme of the expression construct and western blot showing expression of STAG3-EGFP in the STAG3-EGFP HCT116 cells after doxycycline induction for 24 h. Vinculin is shown as loading control. (B) Fractionation of STAG3-EGFP HCT116 cells into soluble and insoluble fractions (chromatin) shows chromatin binding of STAG3-EGFP. Tubulin and histone H3 are shown as loading controls. (C) Co-immunoprecipitation of STAG3-EGFP with SMC3 from STAG3-EGFP-expressing cells shows incorporation of STAG3-EGFP in a chimeric cohesin complex. (D) To test whether STAG3 expression can rescue sister chromatid cohesion in the absence of STAG2, STAG2 was depleted by siRNA knockdown from HCT116 STAG3-EGFP cells, and in parallel, STAG3-EGFP expression was induced. (E) Mitotic chromosome spreads were performed and scored for their morphology. The fractions of the cells in the different categories (normal, separated, scattered) in the different conditions are shown (experiment performed >3 times with similar outcome; here the mean of two independent clones is shown, error bars ± SD; *p* values two-tailed, unpaired *t* test; ∗*p* value ≤0.05, ∗∗*p* value ≤0.01). (F) Cell proliferation of HCT116 STAG3-EGFP cells with and without addition of doxycycline (mean *n* = 6, error bars ± SD, *t* test *p* values). (G) FACS analysis of propidium iodide-stained HCT116 STAG3-EGFP cells without doxycycline and at 24, 48, and 72 h after doxycycline addition was performed (left) to analyze changes in the cell cycle distribution (right) (mean *n* = 2 independent clones, error bars ± SD, *p* values two-tailed, unpaired *t* test,∗*p* value ≤0.05). See also Figure S7.

To test whether STAG3 can substitute for STAG2 but also assess the impact of STAG3 heterologous expression in somatic cells when normal levels of STAG1 and STAG2 are present, we established a HCT116 cell line that allows doxycycline-inducible expression of STAG3-EGFP (Figures 5A and S7A). The overexpressed STAG3 associates with chromatin (Figure 5B) and integrates into the cohesin complex, as shown by co-immunoprecipitation with the cohesin core subunit SMC3 (Figure 5C).

For cells depleted of STAG2, defects in sister chromatid cohesion were reported,^44^^,^^45^ but the mitotic chromosomes of Pt1 and Pt4 fibroblasts appear normal (Figure S3B). Also, STAG3 has been shown to be essential for sister chromatid cohesin during meiosis.^15^ Therefore, we tested whether STAG3 expression can rescue for the sister chromatid cohesion defects seen by small interfering RNA (siRNA)-mediated depletion of STAG2 (Figure 5D). When STAG2 was depleted in uninduced HCT116 STAG3-EGFP dox, we observed in metaphase chromosome spreads an increase of cells that have prematurely lost sister chromatid cohesion (Figure 5E “scattered” fraction) from 11% to 53%. Induction of STAG3-EGFP expression in STAG2-depleted cells reduced the “scattered” fraction to 10%, indicating that STAG3 could substitute for STAG2.

However, overexpression of STAG3 in the presence of STAG2 seems to double the number of prometaphases that have a very mild sister chromatid cohesion defect (from 12% to 24%), observed as slight opening of the primary constriction of the metaphase chromosomes (Figure 5E “separated” fraction). Further, we noticed that HCT116 cells proliferate slower upon induction of STAG3-EGFP (Figure 5F). Fluorescence-activated cell sorting (FACS) analysis of the cell cycle distribution indicated a small enrichment of STAG3-EGFP-positive cells in G1-phase (Figure 5G).

## Discussion

Disease-causing variants identified in *STAG2* are predominantly truncating variants observed in females, although some male cases with missense pathogenic variants are reported.^27^^,^^28^^,^^31^^,^^32^^,^^33^ In our study, we describe six additional females with variants that lead to the presence of only one functional *STAG2* allele. The phenotypic spectrum observed is broad with major congenital abnormalities in all patients, in line with previously published reports. Congenital heart malformations are common in patients with STAG2-truncating variants, and two of our individuals presented with coarctation of the aorta. Left-sided heart lesions, including coarctation of the aorta (this study and^30^) and left ventricular hypoplasia,^30^^,^^34^^,^^46^ as well as VSDs,^27^^,^^46^ were the most frequently observed heart defects in the reported affected individuals so far.

This aligns with findings in female STAG2-knockout mouse embryos, which exhibited severe cardiac defects, including septal abnormalities and malformations of the right ventricle and outflow tract.^47^ These morphological defects may result from altered expression of genes regulating cardiac organogenesis, including heart morphogenesis and remodeling by the secondary heart field progenitor cells. In contrast, patients primarily present with left-sided heart lesions, although septal defects are also common.

Similar to earlier reports on individuals with *STAG2*-truncating variants,^28^ we cannot observe a distinct facial phenotype in our individuals, which makes this cohesinopathy distinct from CdLS. We further corroborate this, since differentially expressed genes (DEGs) observed for STAG2 Pt1 do not correlate strongly with DEGs observed in CdLS cases (Figure S8).^4^

The underlying cause for the phenotypic variation in females with *STAG2*-truncating variants is unclear, but our detailed analysis of primary tissues from individuals with *STAG2*-truncating variants uncovered that skewed X-inactivation might have unexpected contributions to the patient phenotype. Furthermore, remodeled cohesin formed by the integration of somatic and germ cell-specific subunits could also play a role.

In skin fibroblasts of three of four individuals with *STAG2*-truncating variants, STAG2 protein levels were reduced or even absent. The consistent depletion of STAG2-positive cells observed in skin fibroblast samples from anatomically distinct sites in Pt1 indicates that the STAG2 deficiency is likely a generalized feature of the skin. The only skin fibroblast sample of a *STAG2* pathogenic variant (STAG2 p.(Ser327Asn)) reported in the literature did not show altered STAG2 levels.^31^ Strikingly, the remaining fraction of STAG2-positive cells observed in samples from Pt1, Pt3, and Pt4 was reduced over passages, indicating that STAG2-deficient cells outcompete them in culture. Consistent with this, we observed altered expression of cell cycle genes and increased expression of genes linked to translation and ribosome biogenesis that reflect on differences in the fitness of cells.^48^

Studies in mice found that Stag2 deficiency is embryonic-lethal, while Stag2 is dispensable in adult tissues.^47^ However, Stag2 deficiency has an impact on the fitness of some cell types. Stag2-negative cells contributed less to rapidly proliferating tissues like spleen and intestine, but a higher percentage of Stag2-negative cells contributed to brain, pancreas, and liver. During preparation of this manuscript, a study investigating the competition between Stag2 variant cells and wild-type (WT) cells in mouse embryonic development was published.^49^ The Stag2 variants investigated by Buenaventura and coworkers^49^ (amino acid exchanges affecting the Ctcf-Stag2 interaction surface) support the formation of all tissues but were unable to contribute to the lymphoid lineage in the presence of WT cells. Instead, they contributed more to brain and heart relative to WT cells. Unfortunately, neither of these two studies covers skin cells. Whether a similar behavior of human cells underlies the observed heart malformation in Mullegama-Klein-Martinez syndrome individuals remains to be addressed in suitable model systems in a future study.

Our analysis of X inactivation in the cell samples with reduced or absent STAG2 protein showed non-random X inactivation, most likely biased toward inactivation of the intact *STAG2* allele. It is unclear whether and how STAG2 protein levels could affect X inactivation choice during the early phases of human embryonic development. X inactivation in human embryos is less understood but is thought to initiate between the early implantation stage and the end of the first pregnancy month and proceeds progressively, eventually with different kinetics depending on the cell lineage.^50^

In light of the results of Buenaventura et al.^49^ and our observation that STAG2 deficiency might provide a proliferative advantage for skin fibroblasts, the spectrum of observed phenotypes in individuals with STAG2 LoF variants could be driven by a competition between STAG2-positive and -negative cells. To which side the balance between these cells shifts likely depends also on cell-type-specific functions of STAG2.^29^^,^^51^^,^^52^

The most surprising observation of our study was the expression of the germ cell-specific STAG3 paralog in STAG2-deficient but otherwise normal skin fibroblasts. STAG3 overexpression has been previously reported for cancer cells^53^^,^^54^ but not for normal somatic cells. STAG3 can, without the need of other germ cell-specific subunits, integrate into the cohesin complex and associate with chromatin (this study). Other components of the germ cell-specific complex like REC8 depend on STAG3 to integrate in place of RAD21 in a somatic cohesin complex.^55^ Manipulation of STAG3, e.g., depletion by siRNA, was not feasible in the skin fibroblasts. Therefore, we studied the impact of STAG3 overexpression in a HCT116 overexpression model and showed that STAG3 might take over the role of STAG2 for sister chromatid cohesion, similar to its reported role in meiosis.^7^^,^^56^ However, to our knowledge this is the first time that a germ cell-specific protein was shown to functionally replace for its somatic counterpart. Whether STAG3 presence influences the response to STAG2 loss and contributes to the variability of phenotypes across tissues remains an open question that will require future investigation.

### Limitations of the study

Our study was limited by the availability of samples and by the limits of culturing primary skin fibroblasts, like limited passages and very limited options for manipulation of the cells; e.g., transfections were not possible. We decided against the immortalization of the cultures to avoid biases.

However, our observations in the primary tissues, supported by a cell culture model, suggest that females with *STAG2*-truncating variants could present a mosaic pattern of STAG2-positive and STAG2-deficient cells in tissues, with compensatory contributions by the STAG paralog STAG1 and hitherto unknown contribution of STAG3. Additional research is needed to understand whether the observed mosaic expression of STAG2 could be present in different organs and tissues, specifically the systems affected in patients like the brain.

## Resource availability

### Lead contact

The lead contact is Dr. Kerstin S. Wendt (k.wendt@erasmusmc.nl).

### Materials availability

Further information and requests for resources and reagents should be directed to and will be fulfilled by the lead contact Dr. Kerstin S. Wendt (k.wendt@erasmusmc.nl).

### Data and code availability

• Data: Datasets presented in this study were deposited in the Sequencing Read Archive (SRA) SRA:PRJNA1199213 and will become available upon publication of this study.
• Code: No codes were generated specifically for this study.
• Other items: No other items were generated for this study.

## Acknowledgments

The authors thank the affected individuals and their caretakers for participation in this study. We also thank Tim Strom, Elisabeth Graf, and Thomas Schwarzmayr from the Institute of Human Genetics at the Helmholtz Zentrum Muenchen Neuherberg/Germany for RNA sequencing; Rolf Jessberger for antibodies; and Stefan Barakat for input on the manuscript. We also thank the Bachelor and Master students of the K.S.W. lab for help with preparing and analyzing chromosome spreads. Work in the lab of K.S.W. was funded by the Dutch Cancer Society grant EMCR 2015-7857 and by the Netherlands Organisation for Scientific Research (NWO-BBOL) grant 737.016.014.

## Author contributions

M.W.W., M.M.G., V.C., and K.S.W. conceived the study and designed the experiments. V.C., M.M.G., T.v.S., A.H., I.P., and K.S.W. performed experiments. M.W.W., W.K.C., M.W., A.S., I.H., F.J.K., P.T., and M.L. collected clinical data and samples. W.F.J. and J.D. contributed to genomic sequencing and mass spectrometry data and analyses. M.W.W., M.M.G., and K.S.W. analyzed the data. M.M.W., M.M.G., and K.S.W. wrote the manuscript with critical input from I.P., P.T., and W.K.C.

## Declaration of interests

The authors declare that the research was conducted in the absence of any commercial or financial relationships that could be construed as a potential conflict of interest.

## STAR★Methods

### Key resources table

**Table:** 

REAGENT or RESOURCE	SOURCE	IDENTIFIER
**Antibodies**		

Rabbit polyclonal anti-SMC3	Wendt lab	Zuin et al., 2014^57^
Rabbit polyclonal anti-STAG2	Bethyl labs	Cat# A302-580A, RRID:AB_2034860
Goat polyclonal anti-STAG1	Abcam	Cat# AB4457, RRID:AB_2286589
Goat polyclonal anti-STAG1	Bethyl labs	Cat# A300-156A, RRID:AB_185512
Rabbit polyclonal anti-STAG3	Abcam	Cat# AB185109
Rabbit polyclonal anti-STAG3	Sigma-Aldrich	Cat# SAB4500153, RRID:AB_10745135
Rabbit polyclonal anti-STAG3	Rolf Jessberger	–
Mouse monoclonal anti-Phospho-Histone H2A.X (Ser139)	Thermo Fisher Scientific	Cat# 14-9865-80, RRID:AB_2573047
Mouse monoclonal anti-vinculin	Santa Cruz Biotechnology	Cat# sc-59803, RRID:AB_794011
Rabbit polyclonal anti-HP1β	Abcam	Cat# ab10478, RRID:AB_297216
Mouse monoclonal anti-β-tubulin	Sigma-Aldrich	Cat# T8328, RRID:AB_1844090

**Biological samples**		

Patient samples	Contributing clinicians	Table 1

**Chemicals, peptides, and recombinant proteins**		

Puromycin	Thermo Fisher Scientific	Cat#A11138-03;CAS:53-79-2
Doxycycline	Sigma-Aldrich	D9891-5G
Propidium Iodide	Invitrogen	Cat# 15541957; CAS: 25535-16-4

**Critical commercial assays**		

X-tremeGENE™ HP	Roche	6366244001
Cell Counting Kit - 8	Sigma-Aldrich	96992
ReliaPrep RNA Cell Miniprep system	Promega	Z6011
Lipofectamine™ 2000	Thermo Fisher Scientific	11668019
NEXTFlex ChIP-seq kit	BioOScientific	NOVA-5143-01

**Deposited data**		

Raw and analyzed data	This paper	SRA: PRJNA1199213

**Experimental models: Cell lines**		

STAG3-EGFP-HCT116	This paper	N/A
HCT116	ATCC	ATCC® CCL-247
HeLa	ATCC	ATCC® CCL-2, RRID: CVCL_0030

**Oligonucleotides**		

siRNA:	–	–
Control non-targeting control sense CGUACGCGGAAUACUUCGAtt	This paper	N/A
Control non-targeting control antisense UCGAAGUAUUCCGCGUACGtt	This paper	N/A
STAG2 sense AGCACUAACAGAUAGG CAAGAGAGT	This paper	N/A
STAG2 antisense ACUCUCUUGCCU AUCUGUUAGUGCUUC	This paper	N/A

**Recombinant DNA**		

pRTS1	Bornkamm et al.^58^	N/A

**Software and algorithms**		

Bowtie	Langmead et al.^59^	https://bowtie-bio.sourceforge.net/index.shtml
DeepTools	Ramírez et al.^60^	https://deeptools.readthedocs.io/en/latest/
DESeq2	Love et al.^61^	https://bioconductor.org/packages/release/bioc/html/DESeq2.html
FASTQC	Babraham Institute	https://www.bioinformatics.babraham.ac.uk/projects/fastqc/
GSEA	Mootha et al.^62^; Subramanian et al.^63^	https://www.gsea-msigdb.org/gsea/index.jsp
HTseq-count	Anders et al.^64^	https://pypi.org/project/HTSeq/
MACS2	Zhang et al.^65^	https://github.com/macs3-project/MACS/
MaxQuant	Cox and Mann^66^	www.maxquant.org
Metascape	Zhou et al.^67^	https://metascape.org/gp/index.html
Picard	Broad Institute	https://broadinstitute.github.io/picard/
SamTools	Li et al.^68^	https://www.htslib.org/
STAR	Dobin et al.^69^	https://github.com/alexdobin/STAR?tab=readme-ov-file
trimmomatic	Bolger et al.^70^	https://github.com/usadellab/Trimmomatic

### Experimental model and study participant details

#### Selection of affected individuals

After the identification of individual 1 (Pt1) with a truncating *STAG2* variant at the Erasmus University Medical Center Dept. Clinical Genetics, additional cases were identified via our network of collaborators. Individuals were included based on female sex and the presence of variants predicted to cause loss-of-function, as identified through research or routine clinical diagnostics. Affected individuals were examined by their referring physicians and genetic analyses were performed in a diagnostic setting. For Erasmus MC, genome-wide investigations in a diagnostic setting were IRB-approved (METC-2012–387). Probands or their legal guardians gave informed consent for genomic investigations and publication of their anonymized data, including photographs, in accordance with the Declaration of Helsinki.

### Method details

#### X-chromosome inactivation

The degree of X chromosome inactivation (XCI) skewing was assessed by analyzing the methylation status of two polymorphic regions on the X chromosome. Specifically, we examined the highly polymorphic CAG repeat region of the androgen receptor (AR) and the CGG repeat region of the FMR1 gene. For both genes, PCR amplification was performed on undigested DNA and DNA digested with methylation-sensitive enzymes, followed by calculation of allele ratios, as described by Allen et al.^71^ and Carrel and Willard.^72^ Alleles differing by more than two trinucleotide repeats were considered informative.

#### Cell culture

Control fibroblast, patient-derived fibroblasts and HeLa cells were cultured in DMEM supplemented with 10% FBS and 1% Penicillin/Streptomycin and grown at 37°C with 4% CO2. HCT116 cells were cultured in McCoy's 5A medium with 10% FBS and 1% Antibiotics in a cell culture incubator at 37°C with 5% CO2.

#### Generation of STAG3 inducible expression cell line

To establish cell lines that allow inducible expression of STAG3 or STAG3-EGFP, the respective cDNA was cloned into a pRTS1 episomal vector^58^ that allows doxycycline-inducible expression. To generate stable HCT116 cell lines that allow STAG3-EGFP expression, the STAG3-EGFP pRTS1 episomal vector was transfected into the cells using X-tremeGENE™ HP DNA Transfection Reagent (Roche, 6366244001). At 24 hours after transfection selection with 1 μg/ml puromycin was started. After outgrowth of colonies, single colonies were picked and analyzed.

For transient transfection of HeLa cells, the STAG3 pRTS1 episomal vector was transfected without selection.

To induce STAG3 or STAG3-EGFP expression, 2 μg/ml doxycycline was added for 24 hours unless indicated otherwise.

#### Immunoprecipitation

Immunoprecipitation experiments were performed as described (Watrin et al.^73^; Zuin et al.^57^). In brief, immunoprecipitation was performed from control fibroblasts and fibroblasts of Pt1 using anti-SMC3 antibodies. For each IP 4 mg protein extract was used and 10 ug antibodies as well as 10 ul beads (BioRad protein A beads) per 1 mg extract. Immunoprecipitation was performed for 1 h at room temperature and the beads washed 3 times with TBS/T (0,01%TX-100) and twice with TBS/T+250 mM NaCl. Beads were eluted with 100 mM glycine pH 2.

#### Mass spectrometry

Immunoprecipitates were loaded on a NuPAGE Bis-Tris gel and the gel stained with the Colloidal Blue Staining Kit (Invitrogen). SDS-PAGE gel lanes were cut into 2-mm slices and subjected to in-gel reduction with dithiothreitol, alkylation with iodoacetamide and digestion with trypsin (sequencing grade; Promega), as described previously.^74^ Nanoflow liquid chromatography tandem mass spectrometry (nLC-MS/MS) was performed on an EASY-nLC coupled to a Q Exactive mass spectrometer (Thermo) or to an Orbitrap Fusion Tribid mass spectrometer (Thermo), both operating in positive mode. Peptide mixtures were trapped on a ReproSil C18 reversed phase column (Dr Maisch; 1.5 cm × 100 μm) at a rate of 8 μl/min. Peptides were separated on a ReproSil-C18 reversed-phase column (Dr Maisch; 15 cm × 50 μm) using a linear gradient of 0–80% acetonitrile (in 0.1% formic acid) for 170 min at a rate of 200 nl/min. The elution was directly sprayed into the electrospray ionization source of the mass spectrometer. Spectra were acquired in continuum mode; fragmentation of the peptides was performed in data-dependent mode by HCD (Q Exactive) or CID (Orbitrap Fusion).

#### Cell fractionation into soluble and insoluble (chromatin-bound) proteins

To assess the chromatin-bound fraction of proteins, whole-cell extracts of human primary skin fibroblasts were separated into soluble supernatant and chromatin-containing pellet fractions as described.^73^

#### Cell proliferation

Cells were seeded into 96 well plates, 1250 cells per well in 100 ul medium volume. At 24h, 48h, 72h and 96 hours after seeding, 10 ul CCK-8 reagent (Cell Counting Kit – 8, 96992, Sigma-Aldrich) was added to the cells and incubated for 1 h in the cell culture incubator. The reaction was stopped by addition of 10 ul 10% SDS and the colorimetric signal measured with a BioTek Instruments 800TS microplate reader with 450 nm filter.

#### Chromosome spreads

Chromosome spread experiments were performed as described (Parenti et al., 2020). Cells were then spread on a glass slide, dried, and stained with Giemsa stain. Coverslips were mounted with Entellan. Mitotic chromosome spreads were observed using a Olympus BX40 microscope (Olympus, Japan) with a 40x dry objective, with cellSens Entry microscope software.

Images were analysed with ImageJ (version 1.52p, downloaded as Fiji). Telomere cohesion was quantified by measuring the distance between sister telomeres using the PeakFinder Tool. See the results section for more details. For 25 images, 3 telomere distance measurements per image were performed. The samples were blinded during image analysis. Centromere cohesion was also evaluated, as described.^4^

#### ChIP-sequencing

Chromatin immunoprecipitation for SMC3, STAG1 and STAG2 was performed as previously described.^75^^,^^76^ In brief, cells at 70%–80% confluence were crosslinked with 1% formaldehyde for 10 minutes and quenched with 125 mM glycine. After washing with PBS, cells were resuspended in (50 mM Tris-HCl pH 8.0, 1% SDS, 10 mM EDTA, 1 mM and Complete Protease Inhibitor (Roche)) and sonicated (Diagenode Bioruptor, Seraing, Belgium) to around 500 bp. Debris were removed by centrifugation and the lysate was diluted 1:4 with dilution buffer (20 mM Tris-HCl pH 8.0, 0.15 M NaCl, 2 mM EDTA, 1% TX-100, protease inhibitors) and precleared with Affi-Prep Protein A support beads (Bio-Rad). The respective antibodies were incubated with the lysate overnight at 4°C, followed by 2 hours incubation at 4°C with blocked protein A Affiprep beads (Bio-Rad) (blocking solution: 0.1 mg/ml BSA). Beads were washed with washing buffer I (20 mM Tris-HCl pH 8.0, 0.15 M NaCl, 2 mM EDTA, 1% TX-100, 0.1% SDS, 1 mM PMSF), washing buffer II (20 mM Tris-HCl pH 8.0, 0.5 M NaCl, 2 mM EDTA, 1% TX-100, 0.1% SDS, 1 mM PMSF), washing buffer III (10 mM Tris-HCl pH 8.0, 0.25 M LiCl, 1 mM EDTA, 0.5% NP-40, 0.5% sodium deoxycholate) and TE-buffer (10 mM Tris-HCl pH 8.0,1 mM EDTA). Beads were eluted twice (25 mM Tris-HCl pH 7.5, 5 mM EDTA, 0.5% SDS) for 20 minutes at 65°C. The eluates were treated with RNAse H and proteinase K for 1 hour at 37°C and decrosslinked at 65°C overnight. The samples were further purified by phenol-chloroform extraction and ethanol-precipitated. The pellet was dissolved in TE buffer.

#### RNA-sequencing

Fibroblast were grown simultaneously and harvested at identical confluency. We cultured for as few passages as possible. Controls were between 13 and 15 passages total, Pt1 P12 (biopsy1) and P5 (biopsy2), Pt2 P9, Pt3 P10. Cells were harvested and the RNA extracted with the ReliaPrep RNA Cell miniprep System (Promega, Z6011). Strand specific, polyA-enriched RNA sequencing was performed as described earlier (Parenti et al., 2020). For library preparation, 1 μg of RNA was poly(A) selected, fragmented, and reverse transcribed with the Elute, Prime, Fragment Mix (Illumina). A-tailing, adaptor ligation, and library enrichment were performed as described in the TruSeq Stranded mRNA Sample Prep Guide (Illumina). RNA libraries were assessed for quality and quantity with the Agilent 2100 BioAnalyzer and the Quant-iT PicoGreen dsDNA Assay Kit (Life Technologies).

#### Gamma irradiation and γ-H2AX foci count

As described in Parenti et al. (Parenti et al., 2020), patient and control fibroblasts were seeded on a coverslip and exposed to 1 Gy γ-irradiation using a 300kV ceramic X-ray tube (RS320 X-ray machine, Xstrahl). Cells were fixed according to standard immunofluorescence staining protocol and stained with anti-γ-H2AX antibodies (Invitrogen, 14-9865-80) and Alexa Fluor 488-conjugated goat anti-mouse secondary antibodies (Invitrogen, A11029). Images were acquired using an immunofluorescence microscope (Zeiss Imager Z.1). The average number of γ-H2AX foci for at least 50 cells per cell line per time point were counted using ImageJ/FIJI software.

#### Flow cytometric cell cycle analysis

Cells were induced for 24 -, 48 - or 72 hours and thereafter harvested by trypsinization and collected. To fix cells, 70% Ethanol (EtOH) was added dropwise while vortexing cells followed by a 30 minutes incubation at 4°C. Ethanol was removed after spinning down for 5 minutes at 4°C at 1500rpm. Cells were resuspended in PBS containing 0.25 mg/ml RNase A (Purelink,12091021, Invitrogen) and 1μg/ml propidium iodide (BMS500PI, Invitrogen). After a 15 minutes incubation at 37°C, cells were analyzed by flow cytometry (BD, LSRFortessa, BD Biosciences). Lasers with excitations at 488nm and 610nm were used to gain information about the forward scatter (FSC), sideward scatter (SSC), PI signal and EGFP signal. Data is modelled and quantified according to the Watson pragmatic model by BD FACS Diva (BD Biosciences). Graphs and statistical analyses were performed with GraphPad Prism9 (La Jolla, Ca, USA) the cell cycle analyses are presented as mean with n=3-6. A Two way-ANOVA with Dunnett’s multiple comparison test was used to analyse the results, in which a statistical significance was reached with a p value < 0,05. Dunnett’s test is a multiple comparison procedure to compare a number of treatments with a single control, in this case non-induced HCT116 cells.

#### siRNA knockdown of STAG2

The following siRNA oligos (IDT custom design) were used:

STAG2

sense AGCACUAACAGAUAGGCAAGAGAGT

antisense ACUCUCUUGCCUAUCUGUUAGUGCUUC

Control non-targeting

sense CGUACGCGGAAUACUUCGAtt

antisense UCGAAGUAUUCCGCGUACGtt

In brief, HCT116 STAG3-EGFP cells were seeded in a 6 well plate to be 70% confluent at the time of transfection and transfected using Lipofectamine 2000 (Thermo Fisher Scientific, 11668019) according to the manufacturer’s instructions, specifically we used 10 μl Lipofectamine 2000 Reagent together with 100 pmol of the corresponding siRNA duplex. Cells were analyzed 48 hours after transfection.

#### DNA damage repair

Fibroblasts were grown on coverslips and irradiated with a dose of 5 Gy in an xstrahl-RS320. The fibroblasts were fixed for 10 minutes in 4% PFA at RT, washed in PBS and permeabilized for 5 minutes with PBS + 0.1% triton x-100. The permeabilized cells were washed with PBS-T (PBS + 0.01% triton X-100) and kept at 4C in PBS-T + 3% BSA until all timepoints were collected. The cells were then incubated overnight with rabbit anti-STAG2 (Bethyl labs) and mouse anti-γH2A.X (Merck Millipore) in PBS-T 3% BSA at 4C. The 24h timepoint was incubated with primary antibodies for 4 hours. The cells were then washed with PBS-T and incubated with anti-mouse Alexa fluor 488 and anti-rabbit Alexa fluor 594. The coverslips were mounted with Prolong Gold anti-fade reagent and dried overnight.

The slides were analyzed by using a fluorescence microscope (Zeiss Apotome, 63x lens). ImageJ was used to select the nuclei using the DAPI channel and to determine the mean γH2A.X signal intensity and STAG2 signal intensity for each separate nucleus.

### Quantification and statistical analysis

#### ChIP-seq data processing

For sequencing, the DNA libraries were prepared using the NEXTFlex ChIP-Seq kit (BioO Scientific, Austin, TX, USA). These libraries were sequenced according to the Illumina TruSeq v3 protocol on an Illumina HiSeq2500 sequencer (Illumina, San Diego, CA, USA). Single reads were generated of 50 base pairs in length. The quality of DNA sequence was investigated using FASTQC (version 0.11.2), and, when necessary trimmomatic (version 0.32) was used to remove low-quality reads and regions. Quality controlled sequence was aligned to Human genome (hg19) using bowtie (version 1.0.0), and samtools (version 0.1.19) was used to remove reads with mapping quality less than 30, and to keep only aligned reads. Duplicated reads were removed, after alignment, using Picard (version 1.97). For statistics of the ChIP-sequencing see Table S8.

Peaks were called using MACS (macs 2) and peaks were filtered using P value. UCSC tracks were generated after duplicate removal, using deeptools (version 3.0.2). Heatmaps were generated using Deeptools (version 3.0.2).

#### RNA-seq data processing

RNA libraries were sequenced as 150 bp paired-end runs on an Illumina HiSeq4000 platform. The STAR aligner (v 2.4.2a) with modified parameter settings (–twopassMode = Basic) was used for split-read alignment against the human genome assembly hg19 (GRCh37) and UCSC knownGene annotation. To quantify the number of reads mapping to annotated genes we used HTseq-count (v0.6.0). FPKM (Fragments Per Kilobase of transcript per Million fragments mapped) values were calculated using custom scripts. The normalized counts for all datasets are included in Table S7. Differential expression analysis was performed using the R Bioconductor package DESeq2.

Sequencing data information and statistics are shown in Table S8. Genes with P value < 0.05 and an absolute (log2) fold-change > 2 were deemed as differentially expressed and listed in Tables S5 and S6. Volcano plots were generated using R (https://www.R-project.org/).^77^ Gene Ontology and networks were generated through Metascape^67^ and GSEA.^63^

#### Mass spectrometry data processing

Raw mass spectrometry data were analyzed using the MaxQuant software suite (version 1.4.1.2). A false discovery rate of 0.01 for proteins and peptides and a minimum peptide length of seven amino acids were set. The Andromeda search engine was used to search the MS/MS spectra against the Uniprot database (taxonomy: Homo sapiens, release June 2013). A maximum of two missed cleavages was allowed. The peptide tolerance was set to 10 ppm and the fragment ion tolerance was set to 20 mmu for HCD spectra (Q Exactive) or to 0.6 Da for CID spectra (Orbitrap Fusion). The enzyme specificity was set to ‘trypsin’, and cysteine carbamidomethylation was set as a fixed modification. The outputs from MaxQuant are included in Tables S2, S3, and S4.

#### General statistics tests

Information on the statistics tests for the data presented are included in the respective figure legends.

## Supplemental information

Document S1. Figures S1–S8 and Tables S1, S2, and S8

Data S1. Moronta_Wessle_uncropped images_ALL

Table S3. MaxQuant protein groups for SMC3-immunoprecipitation from control (C1) and patient fibroblasts (Pt1), related to STAR Methods

Table S4. MaxQuant evidence table for SMC3-immunoprecipitation from control (C1) and patient fibroblasts (Pt1), related to STAR Methods

Table S5. Differentially expressed genes Pt1 vs. control (*p* value <0.05; log2 FC > 2), related to Figure 3

Table S6. Differentially expressed genes Pt3 vs. control (*p* value <0.05; log2 FC > 2), related to Figure 3

Table S7. Normalized counts of all RNA sequencing samples, related to Figure 3
