# Supplementary material for: STAG2-truncating variants reveal a mosaic STAG2 inactivation pattern and compensatory mechanisms involving cohesin complex remodeling
Source: iScience. 2025 Nov 22;28(12):114195. doi: 10.1016/j.isci.2025.114195 (PMC12765388; doi:10.1016/j.isci.2025.114195)
Supplement: Document S1. Figures S1–S8 and Tables S1, S2, and S8 [file mmc1.pdf]

iScience, Volume *28*

Supplemental information

STAG2-truncating variants reveal a mosaic STAG2 inactivation pattern and compensatory mechanisms involving cohesin complex remodeling

Macarena Moronta Gines, Marja W. Wessels, Valentina Casa, Thomas van Staveren, Amber Hof, Wendy K. Chung, Marjolaine Willems, Anna Sandestig, Irina Huening, Peter Turnpenny, Mathilde Lefebvre, Ilaria Parenti, Frank J. Kaiser, Jeroen Demmers, Wilfred F.J. van Ijcken, and Kerstin S. Wendt

Supplementary information

1. Extended clinical characterization (available for individuals 1 and 3)

2. Supplementary Figures S1-S8

3. Supplementary tables S1-S8

Table S1. Overview experiments performed for the different patient fibroblasts, Related to STAR Methods.

Table S2. Mass spectrometry -Peptide intensity values (unique peptides only) for each STAG ortholog for SMC3-immunoprecipitation from control (C1) and patient fibroblasts (Pt1), Related to STAR Methods.

Table S3. MaxQuant protein groups for SMC3-immunoprecipitation from control (C1) and patient fibroblasts (Pt1), Related to STAR Methods. (separate xls file)

Table S4. MaxQuant evidence table for SMC3-immunoprecipitation from control (C1) and patient fibroblasts (Pt1), Related to STAR Methods. (separate xls file)

Table S5. Differentially expressed genes Pt 1 vs control (P value < 0,05; log2 FC > 2), Related to Figure 3. (separate xls file)

Table S6. Differentially expressed genes Pt 3 vs control (P value < 0,05; log2 FC > 2), related to Figure 3. (separate xls file)

Table S7. Normalized counts of all RNA-seq samples, related to Figure 3. (separate xls file)

Table S8. Sequencing data overview, Related to STAR Methods.

Supplementary references

4. RAW data western blots (separate file)

Uncropped and unprocessed images of the western blots shown in the different figures

Extended clinical characterization:

Individual 1: This female patient was born after an uneventful pregnancy at 40 weeks with a birth weight of 3300 grams 0,5 SD and a Apgar score 8/10. She was the first child from nonconsanguineous parents of Dutch origin with no family history of intellectual disability or congenital abnormalities. She was admitted to the hospital in the first month because of feeding problems and poor growth. Echocardiogram showed coarctation of the aorta, patent ductus arteriosus, small muscular ventricular septal defects (VSDs) and hypertrophic cardiomyopathy. Chromosomal analysis showed a normal female karyotype without 22q11.2 deletion (FISH). Coarctectomy and closure of the patent ductus was performed at the age of six weeks. After surgery, prolonged tube feeding was needed, and feeding problems continued in the first years. Physiotherapy started at the age of nine months as motor development was mildly delayed, at 14 months she started pulling up. Length growth followed the 3th centile. Skeletal age assessment from hand radiograph at 11 years showed 3 to 4 years delay. IGF-1 and IGFBP-3 in blood were repeatedly normal. Head circumference was at 0 SD. No brain MRI was performed. Audiography showed unilateral (left) non-progressive high-tone sensorineural hearing loss of 45 dB. Mild intellectual disability (intelligence quotient 62) was present. Ophthalmologic examination revealed CHRPE (congenital hypertrophy of retinal pigment epithelium) and hypermetropy of +7 diopters.

At 12 years of age clinical genetic re-evaluation was performed and mild dysmorphic features were observed including a stocky build with a relatively wide thorax, short neck with mild webbing and spine lordosis. Numerous melanocytic skin nevi were present, mostly on the back. Facial features included mild upslanting palpebral fissures, mild right eyelid ptosis, and mild asymmetry of the face. Hands showed fetal pads and cutaneous syndactyly III-IV. SNP microarray (Illumina Human Cyto 12) performed on DNA extracted from blood showed a *de novo* heterozygous 160 kb deletion in Xq25 (chrX:122,899,890-123,059,602; NCBI36/hg18), only encompassing the 5' and large part of the *STAG2* gene.

Pubertal development was delayed; breast development and pubic hair was scale Tanner Stage 2 at the age of 15 years. The first menses started at 17 years.

At the age of 20 years, she is working independently at an assembly line. No significant medical problems occurred during the last years. Height followed the 3th centile until 14 years, her final length is 162 cm (-1.4 SD), just under the target height. Target Height-SD 0.36. Echocardiography shows good left -and right ventricular systolic function without *re-coarctation,* a small muscular VSD and minor thickening of the left ventricular septal wall (Figures 1Ai and S1A).

Individual 3: A 6-years-old girl was a third child of non-consanguineous parents. The girl was born at term with a cesarian section after an uneventful pregnancy with a birth weight 3100 g, birth length 47 cm, and head circumference (HC) at birth 35 cm. Her length varied during the first 6 years between -1 SD and -2 SD, weight at -1 SD whereas the head circumference showed a very slowly progressive growth until -2.5 SD at the age of 6 years. Her developmental milestones were delayed: walk debut at the age of 18 months, first words at 2-years of age. The girl presented with an intellectual disability, autistic features, restless behaviour, postnatally developed microcephaly, and skin hypopigmentation with Blaschko's lines pattern almost all over her body, mostly on the chest and the belly. The hair showed dyschromia, partially hypopigmented whereas the rest of it has black color. The girl had some dysmorphic features including temporal narrowing, epicanthus, high palate, markedly posteriorly rotated ears, overfolded helix. Brain MRI showed generalized hypomyelination of the cerebellar subcortical white matter, mostly of the frontal lobes and also involving the juxtracortical u-fibers, subinsular and temporal lobes as well as reduced amount of the white matter subtentorial and hypoplasia of corpus callosum. The deeply localized white matter showed normal myelinization pattern. No demyelination was noticed, no abnormalities of the basal ganglia. The EEG showed moderately abnormal slow basal rhythm without locus changes. SNP-array analysis performed on DNA extracted of both blood and fibroblasts did not show any abnormalities. Single exome sequencing revealed a stop-codon heterozygous gene variant in the STAG2 gene, c.646C>T;p.Arg216*.

The patient has three siblings, two of which are unaffected, while the 8-years-old sister presented with autism and speech delay. She has normal growth and motor development and no inner organs anomalies; no brain MRI changes. Targeted Sanger sequencing of the *STAG2*-gene showed absence of the variant identified in her sister.

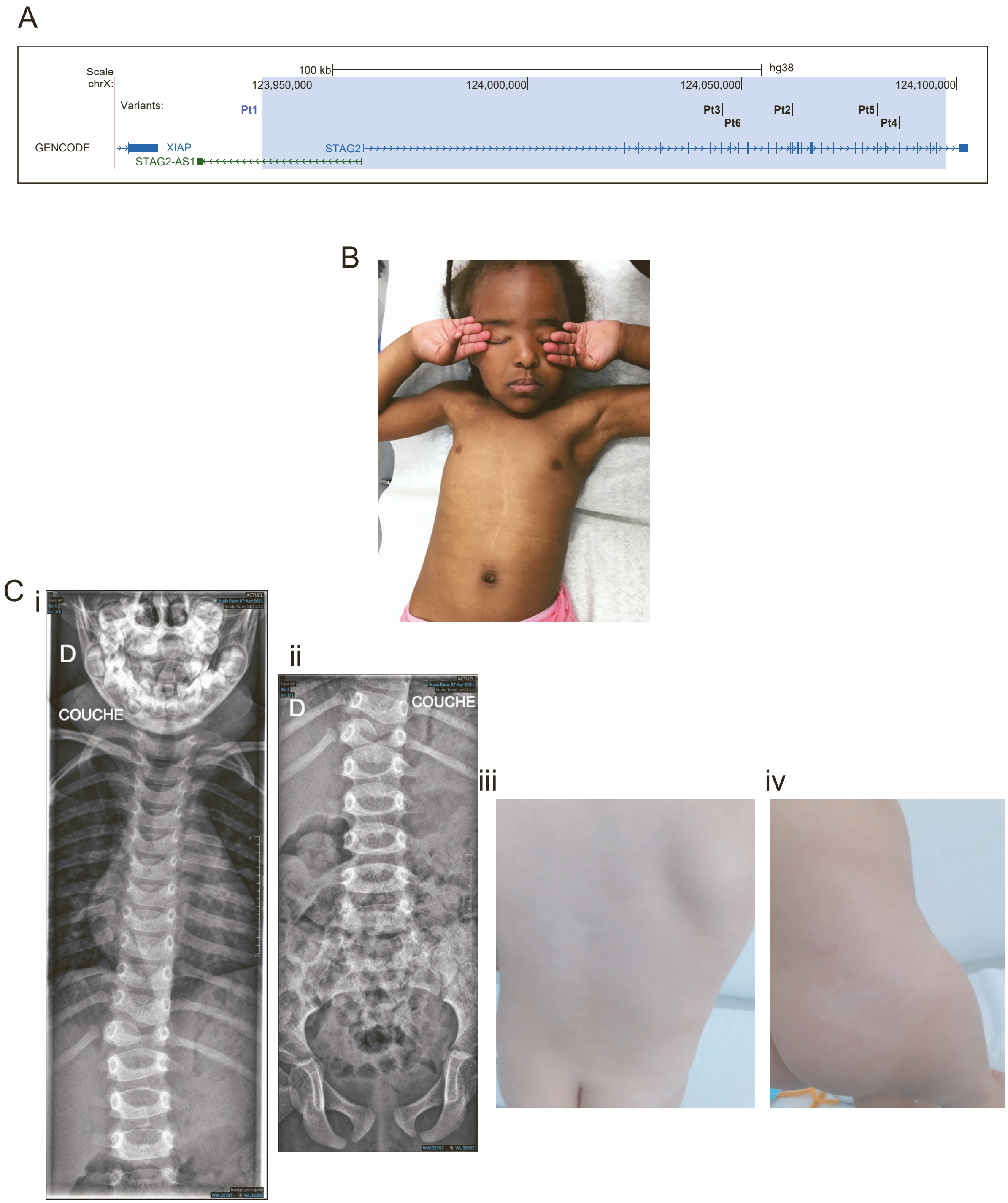

Figure S1. Variants and additional images of Pt3 and Pt4, Related to Figure 1

A) Overview of the genomic locus of the *STAG2* gene generated with the USCS browser (hg38) depicting the deletion found in Pt1 (blue box) and the position of the nonsense variants detected in Pt2, Pt3, Pt4, Pt5 and Pt6.

B) Additional picture of Pt3 showing Blashko lines.

C) Additional pictures of Pt4 showing X-ray of the spine (i and ii), cutis marmorata at the lower back (iii and iv)

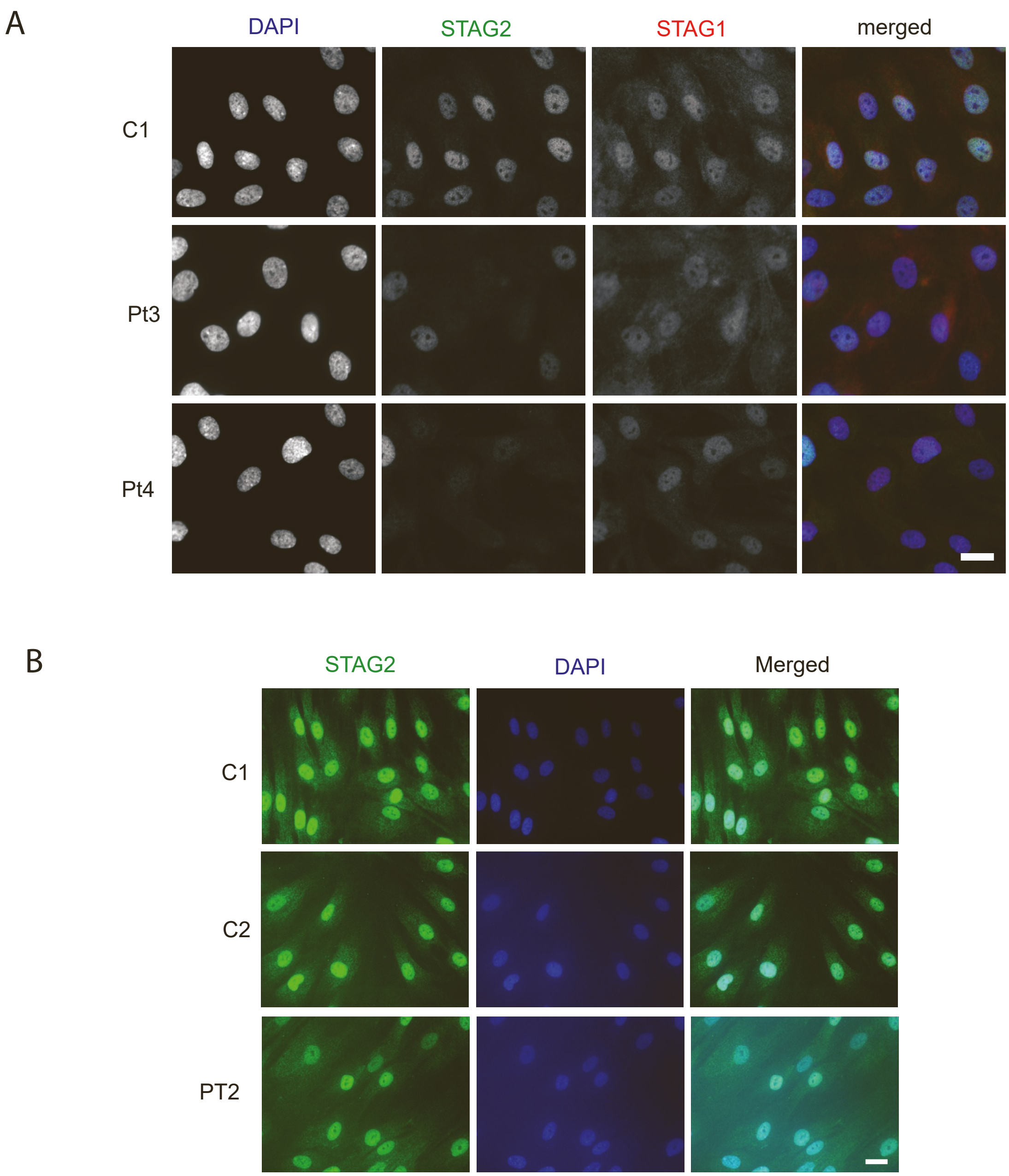

Figure S2.
Immunostaining of different control and STAG2 mutation fibroblasts, Related to Figure1

A) Fibroblasts of C1 and Pt3 and Pt4 were immunostained with antibodies against STAG1 and STAG2. B) Fibroblasts of C1, C2 and Pt2 were immunostained with antibodies against STAG2. DNA was stained using DAPI. Scale bar equals 50 μm.

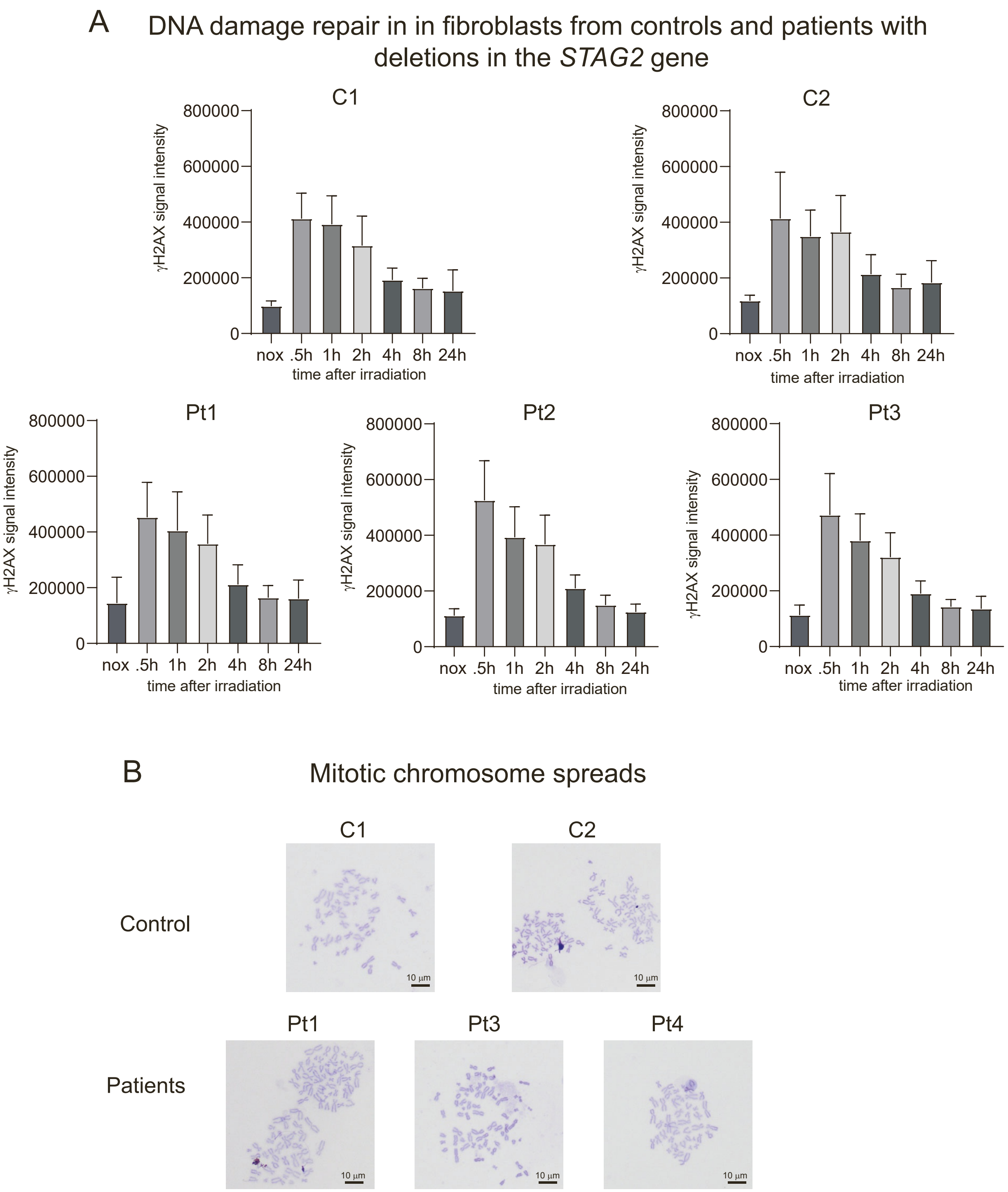

Figure S3.
STAG2-deficient fibroblasts have intact metaphase chromosomes and DNA damage repair Related to Figure 1.
A) DNA damage repair after gamma irradiation was analyzed in fibroblasts of different controls (C1, C2) and patient fibroblasts (Pt1, Pt2 and Pt3) by quantitating the gH2AX signal observed by immunostaining with anti-γH2AX antibodies (mean signal of 80-130 cells analyzed per condition, error bars=+/- s.d.). B) Mitotic chromosome spreads of control and STAG2-deficient (Pt1, Pt3, Pt4) fibroblasts show normal chromosome morphology (>50 spreads were analyzed per cell line). Scale bars equal 10 μm.

Transcript levels (RNA-seq) of cohesin genes in the different fibroblasts

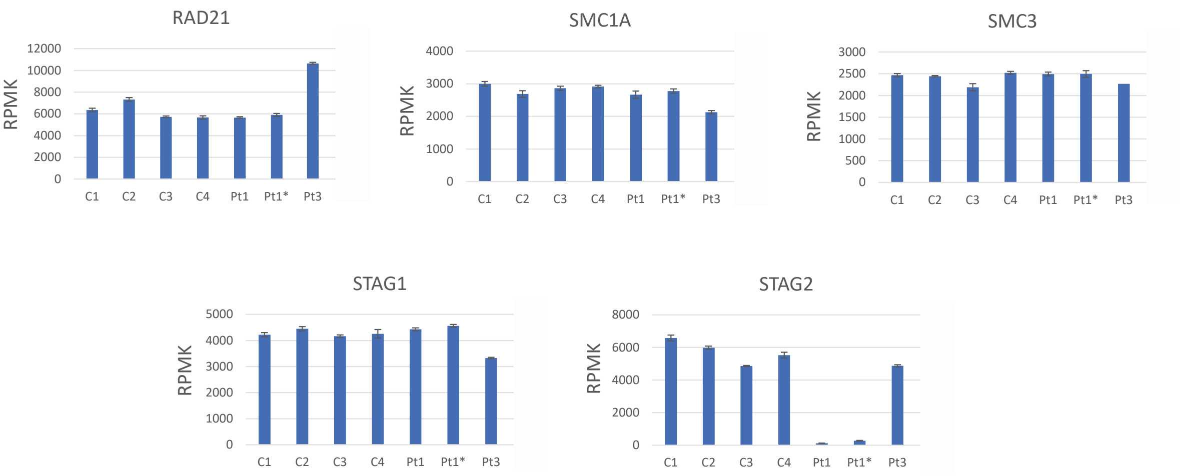

Figure S4.
RNA-seq RPMK data (Table S5) for the control fibroblasts and patient,
Related to Figure 3.
fibroblasts are shown for different cohesin genes (mean of n = 3; error bars +/- s.d.).

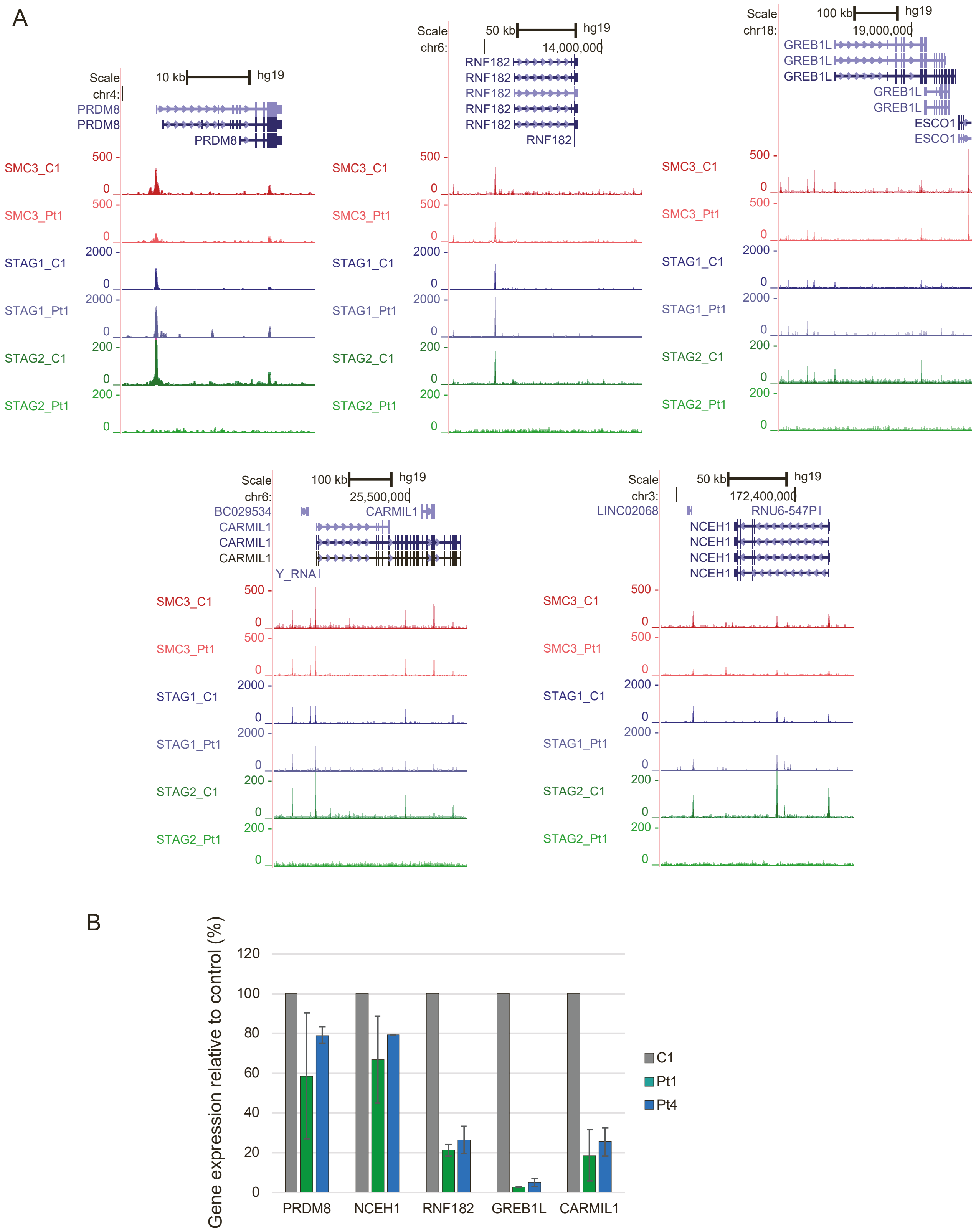

Figure S5. Genes selected for gene expression analysis in Pt4, Related to Figure 3.
A) Binding sites of SMC3, STAG1 and STAG2 close to the gene bodies of the genes.
B) RT-PCR/qPCR analyses of selected genes in control (C1) and patient cells (Pt1 and Pt4) (mean of n = 3; error bars +/- s.d.).

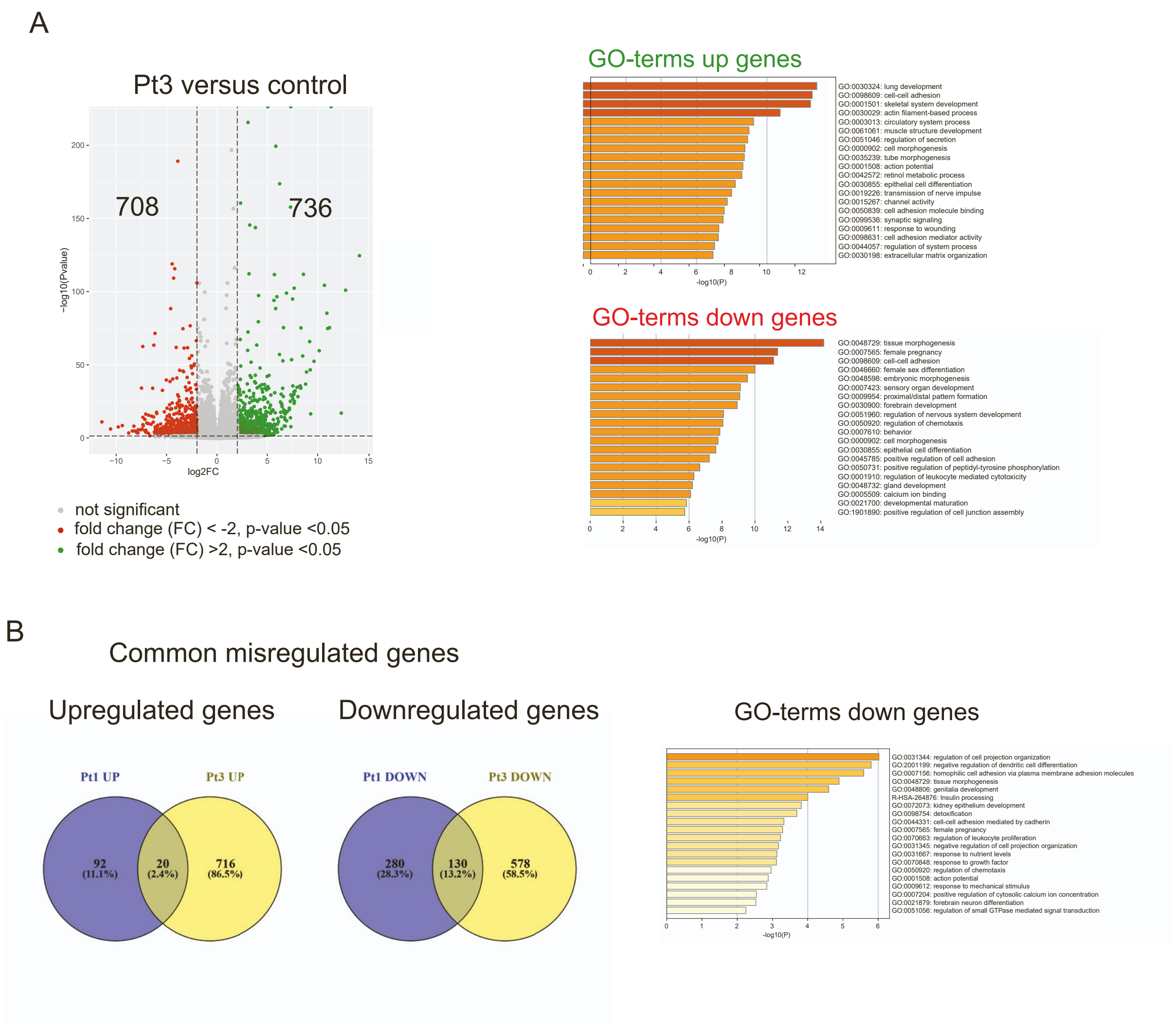

Figure S6. Differentially expressed genes, Related to Figure 3.
Differentially expressed genes in (A) Pt3 are shown as volcano plot and the GO-terms relevant for the respective upregulated or downregulated genes are shown.
(B) The common misregulated genes betweem Pt1 and Pt3 have been clustered in upregulated and downregulated genes.
A GO-term analysis has been performed for the downregulated genes, the number of common upregulated genes was too small to perform a GO-term analysis.

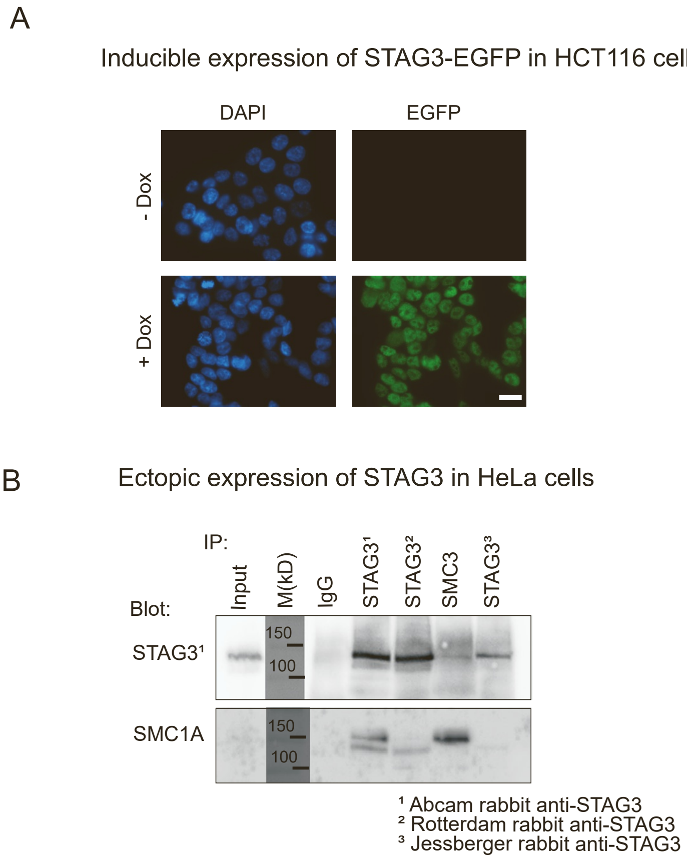

Figure S7.
Cellular models for ectopic expression of STAG3 in cell culture lines, Related to Figure 5.

(A) HCT116 cells that express STAG3-EGFP upon doxycyclin induction were geneated by generating a stable line using STAG3-EGFP pRTS1puro and selection with puromycin. The homogenoeus expression of STAG3 was shown by immunostaining for EGFP. Scale bar equals 50 μm.

(B) STAG3 was expressed ectopically in HeLa cells by transient transfection with the STAG3 pRTS1 episomal plasmid and the cell extracts were used to perform an immunoprecipitation with the different available antibodies against STAG3 and SMC3. Probing the blot for STAG3 and SMC1A indicates that STAG3[1] and STAG3[2] can co-IP cohesin core subunits. The STAG3[1] antibody was used for all western blots and immunostainings in this manuscript.

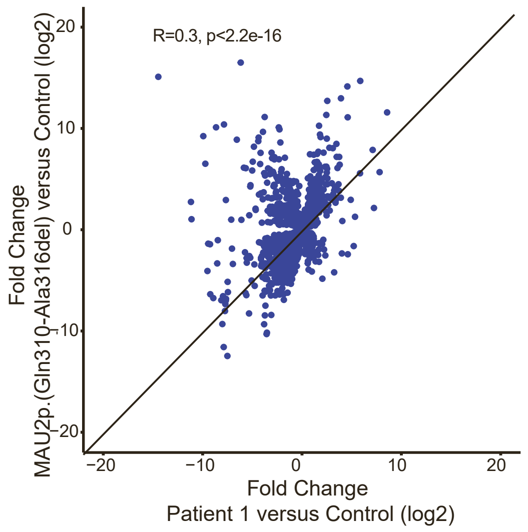

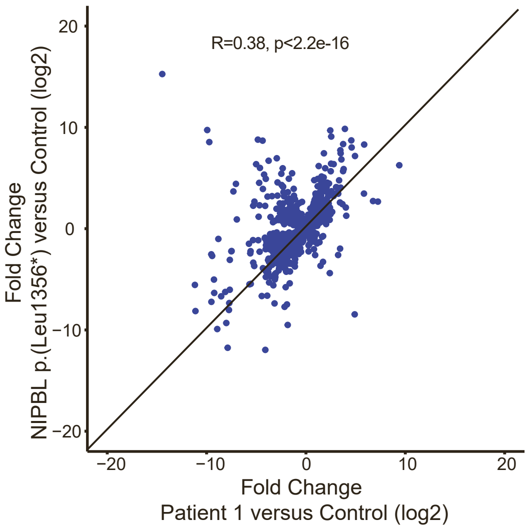

Figure S8. Correlation of differentially expressed genes, Related to discussion.
Correlation of differentially expressed genes between STAG2 Pt1 and two cases of CdLS with NIPBL and MAU2 variants described in Parenti et al., 2020 [1].
Scatter plots showing correlations between the differentially expressed genes observed for STAG2 Pt1 and MAU2 p.(Gln310-Ala316del) or NIPBL p.(Leu1356*). Note, the correlation between MAU2 p.(Gln310-Ala316del) and NIPBL p.(Leu1356*) was R=0.79, p<2.2e-16.

Table S1.
Overview about the experiments performed with the different cell lines, Related to STAR Methods.

	Pt1	Pt2	Pt3	Pt4	Pt5*	Pt6*
Western blot: STAG2	X	X	X	X		
Western blot: STAG3	X	X	X	X		
Immunostaining: STAG2	X	X	X	X		
Immunostaining: STAG3	X					
Proliferation STAG2 pos/STAG2 neg	X		X	X		
Chromosome spreads	X		X	X		
SMC3-IP/Mass spec	X					
RNA-seq	X		X			
ChIP-seq	X					
qPCR validation gene expression changes	X			X		
Chromatin fractionation	X					

*no material available

Table S2.

Peptide intensity values (unique peptide only) for each STAG ortholog, Related to STAR Methods

Immunoprecipitation for SMC3 was performed from control (C1) and patient 1 (Pt1) fibroblasts and analyzed by in-gel digestion and mass spectrometry.

Identified unique peptides for the differnt STAG orhologs are listed for the control (red) and P1 (blue), together with intensity scores on a color scale from orange (high intensity - dark orange, low intensity-white).

Unambiguously identified peptides are listed at the bottom of the table.

Sample	Gene names	Peptide sequence	Intensity
Patient_SMC3	STAG3	LTEHLIPLLPQLLAK	1217400
Patient_SMC3	STAG3	GLASAAGEFLYWK	736990
Patient_SMC3	STAG3	ALSASSSSSASLPFDDR	671470
Patient_SMC3	STAG3	FYNDYGDIIK	643810
Patient_SMC3	STAG3	EYDVAVEAVR	578560
Patient_SMC3	STAG3	HLELFLQQLQEVVVK	215390
Control_SMC3	STAG1;DKFZp781D1416	TDTMIQTPGPLPAPQLTSTVLR	5262500
Control_SMC3	STAG1;DKFZp781D1416	LIIYDIVDMHAAADIFK	3088100
Control_SMC3	STAG1;DKFZp781D1416	ITDGSPSKEDLLVLR	2256800
Control_SMC3	STAG1;DKFZp781D1416	NSLVTGGEDDR	2214200
Control_SMC3	STAG1;DKFZp781D1416	EYDVAVEAIR	1792200
Control_SMC3	STAG1;DKFZp781D1416	LTEHFIITLPMLLSK	1572600
Control_SMC3	STAG1;DKFZp781D1416	REDVWLPLISYR	1441000
Control_SMC3	STAG1;DKFZp781D1416	LELFTNR	1273100
Control_SMC3	STAG1;DKFZp781D1416	VEDESLDNTWLNR	980930
Control_SMC3	STAG1;DKFZp781D1416	EDVWLPLISYR	714180
Patient_SMC3	STAG1;DKFZp781D1416	NSLVTGGEDDR	19980000
Patient_SMC3	STAG1;DKFZp781D1416	HDPQAEEALAK	19744000
Patient_SMC3	STAG1;DKFZp781D1416	TDTMIQTPGPLPAPQLTSTVLR	18217000
Patient_SMC3	STAG1;DKFZp781D1416	SQLIDEFVDR	10821000
Patient_SMC3	STAG1;DKFZp781D1416	ITDGSPSKEDLLVLR	7949900
Patient_SMC3	STAG1;DKFZp781D1416	LIIYDIVDMHAAADIFK	7874700
Patient_SMC3	STAG1	MITSELPVLQDSTNETTAHSDAGSE	7788600
Patient_SMC3	STAG1;DKFZp781D1416	NSLVTGGEDDR	6657900
Patient_SMC3	STAG1	NMQNAEIIR	6349800
Patient_SMC3	STAG1;DKFZp781D1416	REDVWLPLISYR	6101500
Patient_SMC3	STAG1;DKFZp781D1416	IVSMTLDKEYDVAVEAIR	5984800
Patient_SMC3	STAG1;DKFZp781D1416	EYDVAVEAIR	5895800
Patient_SMC3	STAG1;DKFZp781D1416	RVEDESLDNTWLNR	5830900
Patient_SMC3	STAG1;DKFZp781D1416	LELFTNR	5551000
Patient_SMC3	STAG1;DKFZp781D1416	ALQSLYTNR	5343100
Patient_SMC3	STAG1;DKFZp781D1416	ITDGSPSKEDLLVLR	4394300
Patient_SMC3	STAG1;DKFZp781D1416	VEDESLDNTWLNR	4256600
Patient_SMC3	STAG1;DKFZp781D1416	ALQSLYTNR	4254500
Patient_SMC3	STAG1;DKFZp781D1416	LTEHFIITLPMLLSK	4160000
Patient_SMC3	STAG1;DKFZp781D1416	TYSILCSEEYTIQNR	3871100
Patient_SMC3	STAG1;DKFZp781D1416	TDTMIQTPGPLPAPQLTSTVLR	3868700
Patient_SMC3	STAG1;DKFZp781D1416	ELQENQDEIENMMNSIFK	3656400
Patient_SMC3	STAG1;DKFZp781D1416	LIIYDIVDMHAAADIFK	3382800

Patient_SMC3	STAG1;DKFZp781D1416	GLMEEDAEPIFEDVMMSSR	3095500
Patient_SMC3	STAG1;DKFZp781D1416	TDTMIQTPGPLPAPQLTSTVLR	2945700
Patient_SMC3	STAG1;DKFZp781D1416	LELFTNR	2894700
Patient_SMC3	STAG1;DKFZp781D1416	HLDALLK	2832800
Patient_SMC3	STAG1;DKFZp781D1416	ELQENQDEIENMMNSIFK	2831300
Patient_SMC3	STAG1;DKFZp781D1416	YVGWTLHDR	2737100
Patient_SMC3	STAG1;DKFZp781D1416	HVESDVLEACSK	2401600
Patient_SMC3	STAG1;DKFZp781D1416	REDVWLPLISYR	2325400
Patient_SMC3	STAG1	ANGHPQQNGEGEPVTLFEVVK	2296700
Patient_SMC3	STAG1;DKFZp781D1416	EDVWLPLISYR	2113400
Patient_SMC3	STAG1;DKFZp781D1416	EDLLVLR	1983500
Patient_SMC3	STAG1;DKFZp781D1416	ELQENQDEIENMMNSIFK	1981000
Patient_SMC3	STAG1;DKFZp781D1416	ELQENQDEIENMMNSIFK	1955600
Patient_SMC3	STAG1;DKFZp781D1416	ELQENQDEIENMMNSIFK	1800000
Patient_SMC3	STAG1;DKFZp781D1416	VEDESLDNTWLNR	1793300
Patient_SMC3	STAG1;DKFZp781D1416	YVGWTLHDR	1777000
Patient_SMC3	STAG1;DKFZp781D1416	LTEHFIITLPMLLSK	1726000
Patient_SMC3	STAG1;DKFZp781D1416	ELQENQDEIENMMNSIFK	1562900
Patient_SMC3	STAG1	NMQNAEIIR	1358900
Patient_SMC3	STAG1;DKFZp781D1416	REDVWLPLISYR	1201200
Patient_SMC3	STAG1	KMTEEFDEDSGDYPLTMPGPQWK	1158500
Patient_SMC3	STAG1;DKFZp781D1416	LIIYDIVDMHAAADIFK	1127600
Patient_SMC3	STAG1;DKFZp781D1416	FLTEQMMER	1081900
Patient_SMC3	STAG1;DKFZp781D1416	EDVWLPLISYR	867120
Patient_SMC3	STAG1;DKFZp781D1416	ITDGSPSKEDLLVLR	692580
Patient_SMC3	STAG1;DKFZp781D1416	ELQENQDEIENMMNSIFK	644090
Patient_SMC3	STAG1;DKFZp781D1416	LIIYDIVDMHAAADIFK	585620
Patient_SMC3	STAG1;DKFZp781D1416	AICIEEIGVWMK	566690
Patient_SMC3	STAG1;DKFZp781D1416	PVAVAAGEFLHK	375200
Patient_SMC3	STAG1;DKFZp781D1416	LTEHFIITLPMLLSK	339790
Patient_SMC3	STAG1;DKFZp781D1416	RNLLAAFSK	333310
Patient_SMC3	STAG1;DKFZp781D1416	FLTEQMMER	285660
Patient_SMC3	STAG1;DKFZp781D1416	LTEHFIITLPMLLSK	224040
Patient_SMC3	STAG1;DKFZp781D1416	HDPQAEEALAK	
Patient_SMC3	STAG1;DKFZp781D1416	HDPQAEEALAK	
Control_SMC3	STAG2	MNGHHQQNGVENMMLFEVVK	38399000
Control_SMC3	STAG2	LRPEDSFMSVYPMQTEHHQTPLDY	15439000
Control_SMC3	STAG2	LRPEDSFMSVYPMQTEHHQTPLDY	12585000
Control_SMC3	STAG2	EQTLHTPVMMQTPQLTSTIMR	10481000
Control_SMC3	STAG2	NSLLAGGDDDTMSVISGISSR	8257400
Control_SMC3	STAG2	FALTFGLDQLK	8240800
Control_SMC3	STAG2	SQLIDELADK	6535500
Control_SMC3	STAG2	IVSMTLDKEYDVAVQAIK	5502400
Control_SMC3	STAG2	SQLIDELADKFNR	5100200
Control_SMC3	STAG2	TVYVYLEK	4608300
Control_SMC3	STAG2	TYHALCNEEFTIFNR	4365000
Control_SMC3	STAG2	EYDVAVQAIK	3665800
Control_SMC3	STAG2	NSLLAGGDDDTMSVISGISSR	2900100

Control_SMC3	STAG2	LLEDFLQEGEEPDEDDAYQVLSTLK	2646800
Control_SMC3	STAG2	ITAFHNAHDLSK	2467000
Control_SMC3	STAG2	ELQENQDEIENMMNAIFK	2354500
Control_SMC3	STAG2	HMQNSEIIR	2264900
Control_SMC3	STAG2	FALTFGLDQLK	2239400
Control_SMC3	STAG2	ELQENQDEIENMMNAIFK	1873700
Control_SMC3	STAG2	GVVTAEMFR	1871600
Control_SMC3	STAG2	FMTFQMSLR	1782700
Control_SMC3	STAG2	FMTFQMSLR	1655300
Control_SMC3	STAG2	HTDTDVLEACSK	1602600
Control_SMC3	STAG2	ERTELKPDFFDPASIMDESVLGVSM	1270800
Control_SMC3	STAG2	EDVWLPLMSYR	1186400
Control_SMC3	STAG2	GVVTAEMFR	1152900
Control_SMC3	STAG2	LIVYTVVEMNTAADIFK	1030000
Control_SMC3	STAG2	GVVTAEMFR	1002000
Control_SMC3	STAG2	FALTFGLDQLK	805590
Control_SMC3	STAG2	EQTLHTPVMMQTPQLTSTIMR	496120
Control_SMC3	STAG2	HLDALLR	
Patient_SMC3	STAG2	FALTFGLDQLK	8053800
Patient_SMC3	STAG2	FALTFGLDQLK	2840100
Patient_SMC3	STAG2	RFALTFGLDQLK	1589800
Patient_SMC3	STAG2	RFALTFGLDQLK	1571800
Patient_SMC3	STAG2	FALTFGLDQLK	371140
Control_SMC3	STAG2;STAG1	YYNDYGDIIK	8863800
Control_SMC3	STAG2;STAG1;DKFZp781D141	MYSDAFLNDSYLK	8358300
Control_SMC3	STAG2;STAG1	SAMQSVVDDWIESYK	7512700
Control_SMC3	STAG2;STAG1;DKFZp781D141	LELLLQK	6015200
Control_SMC3	STAG2;STAG1;DKFZp781D141	MYSDAFLNDSYLK	3948600
Control_SMC3	STAG2;STAG1	YYNDYGDIIK	3821400
Control_SMC3	STAG2;STAG1	SAMQSVVDDWIESYK	2359000
Control_SMC3	STAG2;STAG1	SAMQSVVDDWIESYK	1720100
Control_SMC3	STAG2;STAG1	SAMQSVVDDWIESYK	911250
Control_SMC3	STAG2;STAG1	YYNDYGDIIK	442980
Control_SMC3	STAG3;STAG2	LELFTSR	3252500
Patient_SMC3	STAG2;STAG1	SAMQSVVDDWIESYK	7639900
Patient_SMC3	STAG2;STAG1;DKFZp781D141	MYSDAFLNDSYLK	7159200
Patient_SMC3	STAG2;STAG1	YYNDYGDIIK	6137600
Patient_SMC3	STAG2;STAG1;DKFZp781D141	LELLLQK	5580000
Patient_SMC3	STAG2;STAG1	YYNDYGDIIK	4557300
Patient_SMC3	STAG2;STAG1;DKFZp781D141	DGIEFAFK	3715600
Patient_SMC3	STAG2;STAG1;DKFZp781D141	LELLLQK	3347300
Patient_SMC3	STAG2;STAG1;DKFZp781D141	MYSDAFLNDSYLK	3255600
Patient_SMC3	STAG2;STAG1;DKFZp781D141	MYSDAFLNDSYLK	3059100
Patient_SMC3	STAG2;STAG1	SAMQSVVDDWIESYK	2045700
Patient_SMC3	STAG2;STAG1	SAMQSVVDDWIESYK	1300900
Patient_SMC3	STAG2;STAG1	SAMQSVVDDWIESYK	816660

Table S8. Sequencing data statistics, related to STAR Methods

RNA-sequencing samples including cell passage number:

Cells (passage)	Replicate	Sample	Total reads	Mapped reads
Control 1 (p15)	Rep1	LUEB0131_S1_R1_CTRL_FIBRO_86E1373_mrg,fastq<br>LUEB0131_S1_R2_CTRL_FIBRO_86E1373_mrg,fastq	98396642	95797240 (97.36%)
Control 1 (p15)	Rep2	LUEB0132_S2_R1_CTRL_FIBRO_86E1373_mrg,fastq<br>LUEB0132_S2_R2_CTRL_FIBRO_86E1373_mrg,fastq	110244507	107560603 (97.57%)
Control 1 (p15)	Rep3	LUEB0133_S3_R1_CTRL_FIBRO_86E1373_mrg,fastq<br>LUEB0133_S3_R2_CTRL_FIBRO_86E1373_mrg,fastq	109867304	107182736 (97.56%)
Control 2 (p14)	Rep1	LUEB0137_S7_R1_CTRL_US_NTO11_mrg,fastq<br>LUEB0137_S7_R2_CTRL_US_NTO11_mrg,fastq	111708361	108994838 (97.57%)
Control 2 (p14)	Rep2	LUEB0138_S8_R1_CTRL_US_NTO11_mrg,fastq<br>LUEB0138_S8_R2_CTRL_US_NTO11_mrg,fastq	109254394	106832982 (97.78%)
Control 2 (p14)	Rep3	LUEB0139_S9_R1_CTRL_US_NTO11_mrg,fastq<br>LUEB0139_S9_R2_CTRL_US_NTO11_mrg,fastq	107381725	104463096 (97.28%)
Control 3 (p13)	Rep1	LUEB0140_S10_R1_CTRL2_HL_mrg,fastq<br>LUEB0140_S10_R2_CTRL2_HL_mrg,fastq	106824292	104201199 (97.54%)
Control 3 (p13)	Rep2	LUEB0141_S11_R1_CTRL2_HL_mrg,fastq<br>LUEB0141_S11_R2_CTRL2_HL_mrg,fastq	94637823	92587178 (97.83%)
Control 3 (p13)	Rep3	LUEB0142_S12_R1_CTRL2_HL_mrg,fastq<br>LUEB0142_S12_R2_CTRL2_HL_mrg,fastq	110229027	107447802 (97.48%)
Control 4 (p13)	Rep1	LUEB0146_S16_R1_CTRL5_HL_mrg,fastq<br>LUEB0146_S16_R2_CTRL5_HL_mrg,fastq	126195665	120897131 (95.80%)
Control 4 (p13)	Rep2	LUEB0147_S17_R1_CTRL5_HL_mrg,fastq<br>LUEB0147_S17_R2_CTRL5_HL_mrg,fastq	117652425	112988603 (96.04%)
Control 4 (p13)	Rep3	LUEB0148_S18_R1_CTRL5_HL_mrg,fastq<br>LUEB0148_S18_R2_CTRL5_HL_mrg,fastq	111401153	106708768 (95.79%)
Patient 1<br>Biosy 1 (p12)	Rep1	LUEB0149_S19_R1_delSA2_RDAM_I_mrg,fastq<br>LUEB0149_S19_R2_delSA2_RDAM_I_mrg,fastq	111082932	107314785 (96.61%)
Patient 1<br>Biosy 1 (p12)	Rep2	LUEB0150_S20_R1_delSA2_RDAM_I_mrg,fastq<br>LUEB0150_S20_R2_delSA2_RDAM_I_mrg,fastq	114925237	110654091 (96.28%)
Patient 1<br>Biosy 1 (p12)	Rep3	LUEB0151_S21_R1_delSA2_RDAM_I_mrg,fastq<br>LUEB0151_S21_R2_delSA2_RDAM_I_mrg,fastq	98115762	94234097 (96.04%)
Patient 1<br>Biosy 2 (p5)	Rep1	LUEB0152_S22_R1_delSA2_RDAM_II_mrg,fastq<br>LUEB0152_S22_R2_delSA2_RDAM_II_mrg,fastq	107948356	104041052 (96.38%)
Patient 1<br>Biosy 2 (p5)	Rep2	LUEB0153_S23_R1_delSA2_RDAM_II_mrg,fastq<br>LUEB0153_S23_R2_delSA2_RDAM_II_mrg,fastq	112897892	109081235 (96.62%)
Patient 1<br>Biosy 2 (p5)	Rep3	LUEB0154_S24_R1_delSA2_RDAM_II_mrg,fastq<br>LUEB0154_S24_R2_delSA2_RDAM_II_mrg,fastq	112486009	108123051 (96.12%)
Patient 3 (p10)	Rep1	LUEB0134_S4_R1_delSA2_HL_I_mrg,fastq<br>LUEB0134_S4_R2_delSA2_HL_I_mrg,fastq	105648432	103057993 (97.55%)
Patient 3 (p10)	Rep2	LUEB0135_S5_R1_delSA2_HL_I_mrg,fastq<br>LUEB0135_S5_R2_delSA2_HL_I_mrg,fastq	109205105	106677074 (97.69%)
Patient 3 (p10)	Rep3	LUEB0136_S6_R1_delSA2_HL_I_mrg,fastq<br>LUEB0136_S6_R2_delSA2_HL_I_mrg,fastq	115660982	112809054 (97.53%)

ChIP-sequencing

Cells	Antibody	Sample	Total Reads	Mapped Reads
Control 1 fibroblasts	SMC3	SMC3_control_L002_R1_001,fastq	10548686	9434616 (89.44%)
Control 1 fibroblasts	STAG1	STAG1_control_S22_L001_R1_001,fastq	3587271	3430444 (95.63%)
Control 1 fibroblasts	STAG2	STAG2_control_L002_R1_001,fastq	32557623	30852968 (94.76%)
Pt1 fibroblasts	SMC3	SMC3_STAG2D_L002_R1_001,fastq	20786016	17954729 (86.38%)
Pt1 fibroblasts	STAG1	STAG1_STAG2D_S23_L001_R1_001,fastq	2123183	2010432 (94.69%)
Pt1 fibroblasts	STAG2	STAG2_STAG2D_L002_R1_001,fastq	28124961	26986650 (95.95%)

Supplementary reference:

1. I. Parenti, F. Diab, S. R. Gil, E. Mulugeta, V. Casa, R. Berutti, R. W. W. Brouwer, V. Dupe, J. Eckhold, E. Graf, et al. (2020). MAU2 and NIPBL Variants Impair the Heterodimerization of the Cohesin Loader Subunits and Cause Cornelia de Lange Syndrome. Cell Rep *31*, 107647. 10.1016/j.celrep.2020.107647.
