# Supplementary material for: STAG2-truncating variants reveal a mosaic STAG2 inactivation pattern and compensatory mechanisms involving cohesin complex remodeling
Source: iScience. 2025 Nov 22;28(12):114195. doi: 10.1016/j.isci.2025.114195 (PMC12765388; doi:10.1016/j.isci.2025.114195)
Supplement: Data S1. Moronta_Wessle_uncropped images_ALL [file mmc2.pdf]

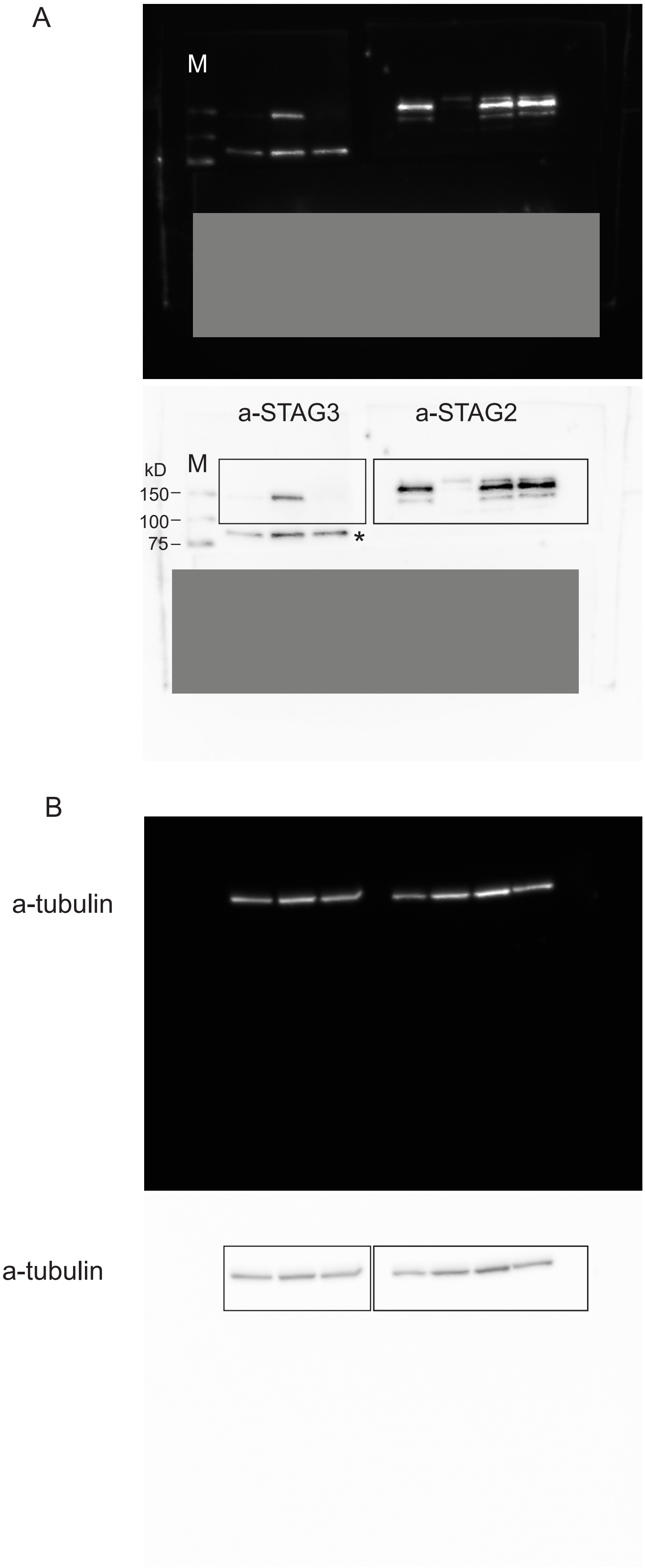

Figs 1B and 4B

A) Original exposure of the two sections of a membroane probed with either anti-STAG3 and anti-STAG2. The cut membrane was imaged side-by-side and the grey box coveres a membrane belonging to another project. For the figure the signal was inverted to make the bands better visible. The parts used for the figures are indicated with boxes. Note that the anti-STAG3 antibody shows an unspecific signal(*).

B) The lover part of the membrane shown in (A) was probed with anti-tubulin.

A

a-STAG2
1:1000
Bethyl labs

a-tubulin
1:1000

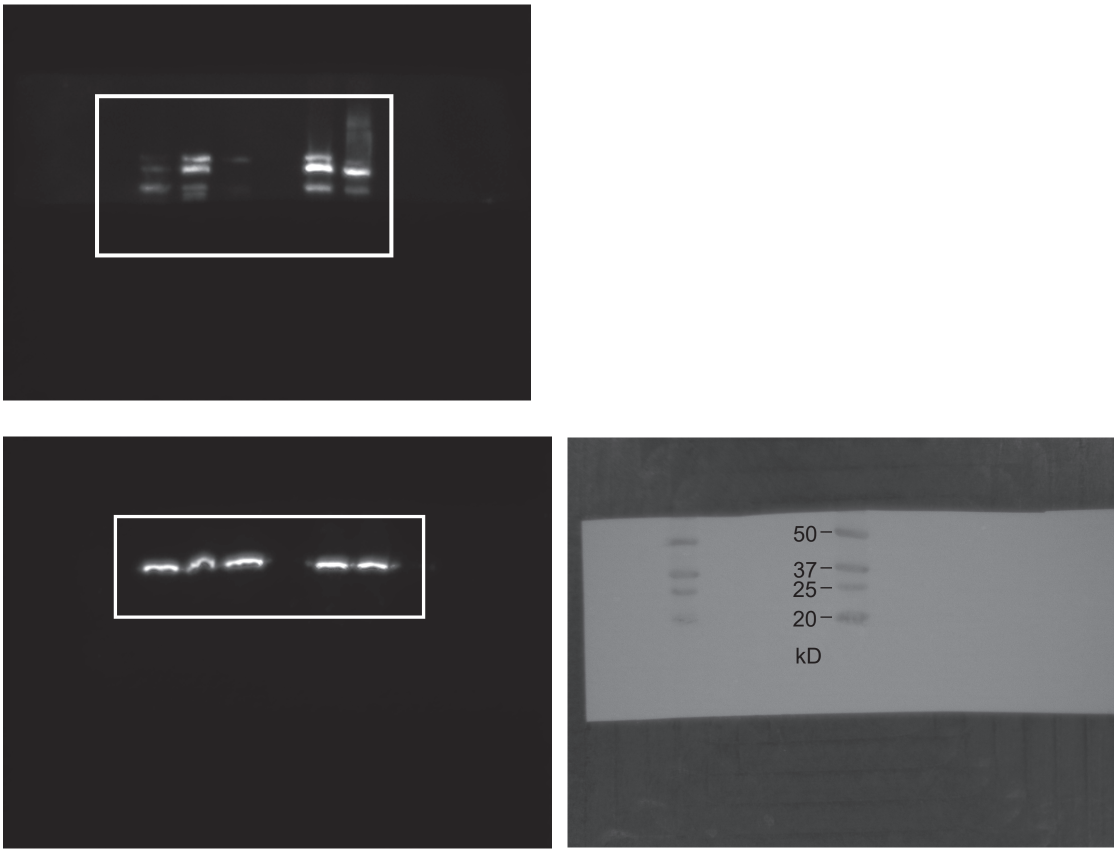

Fibroblasts LCL

C1 Pt4 C4 Pt1

B

a-STAG2
1:1000
Bethyl labs

a-tubulin
1:1000

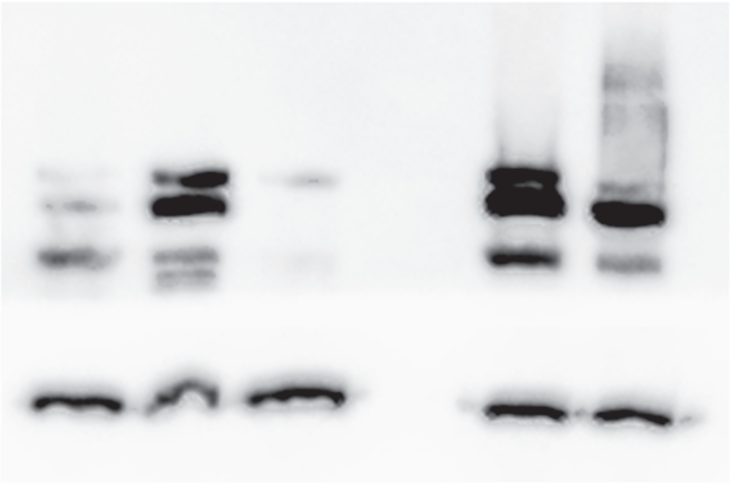

Fig 1F

A)

Raw images with the parts used in the figure indicated

B)

Image as presented in the figure.

The signals of the blots were inverted to allow a better appreciation of the signals.

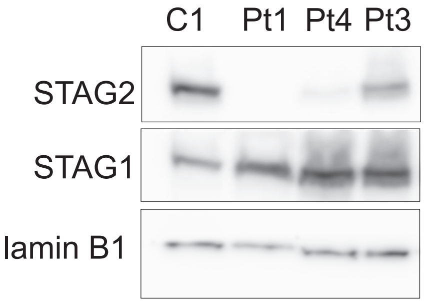

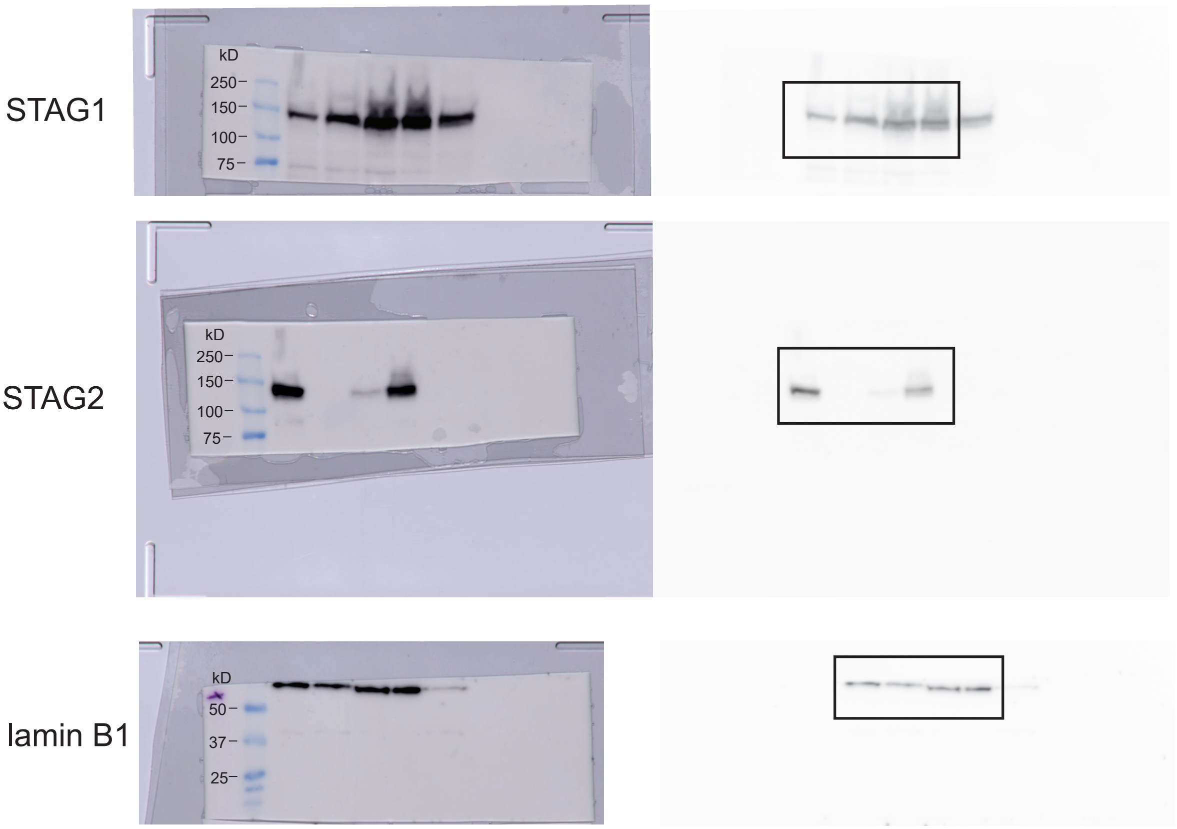

Fig 1G

The left side shows a long exposure of the membrane overlayed with the marker.

A shorter exposure was used to assemble the figure (right side). The parts used for the figure are indicated with boxes. Note that the menbrane was first probed with mouse anti-STAG2 and after quenching of the signal with goat anti-STAG1.

Also note that the blot was reprobed with rabbit anti-STAG3 and anti-tubulin for Fig. 4B.

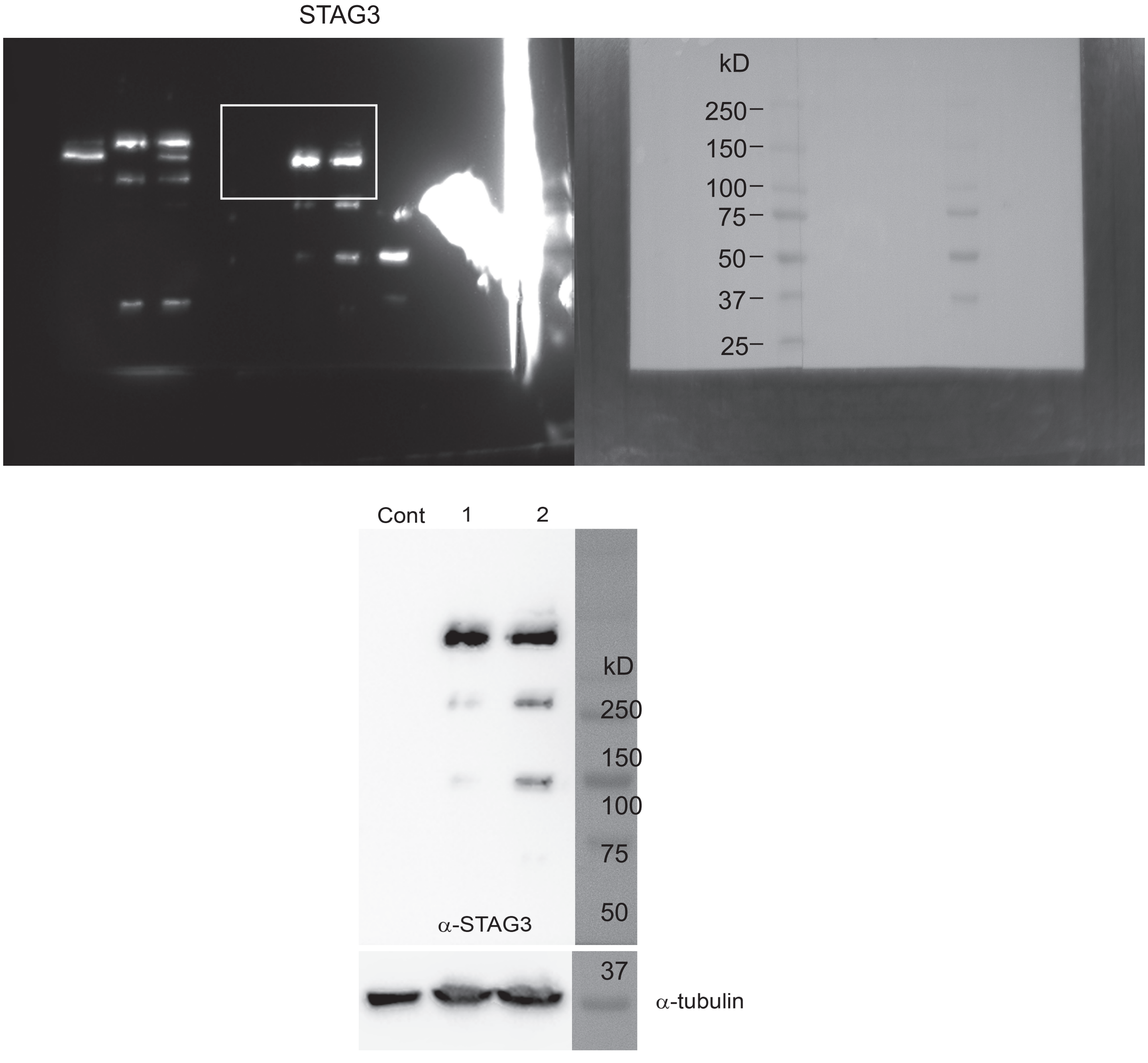

Fig 4A
The upper part shows the raw image with the section used for the figure marked with a box.
And the image of the blot with markers.
Below is an overlay of the blot with the marker. The signal of the blot was inverted to allow better visibility of the bands.

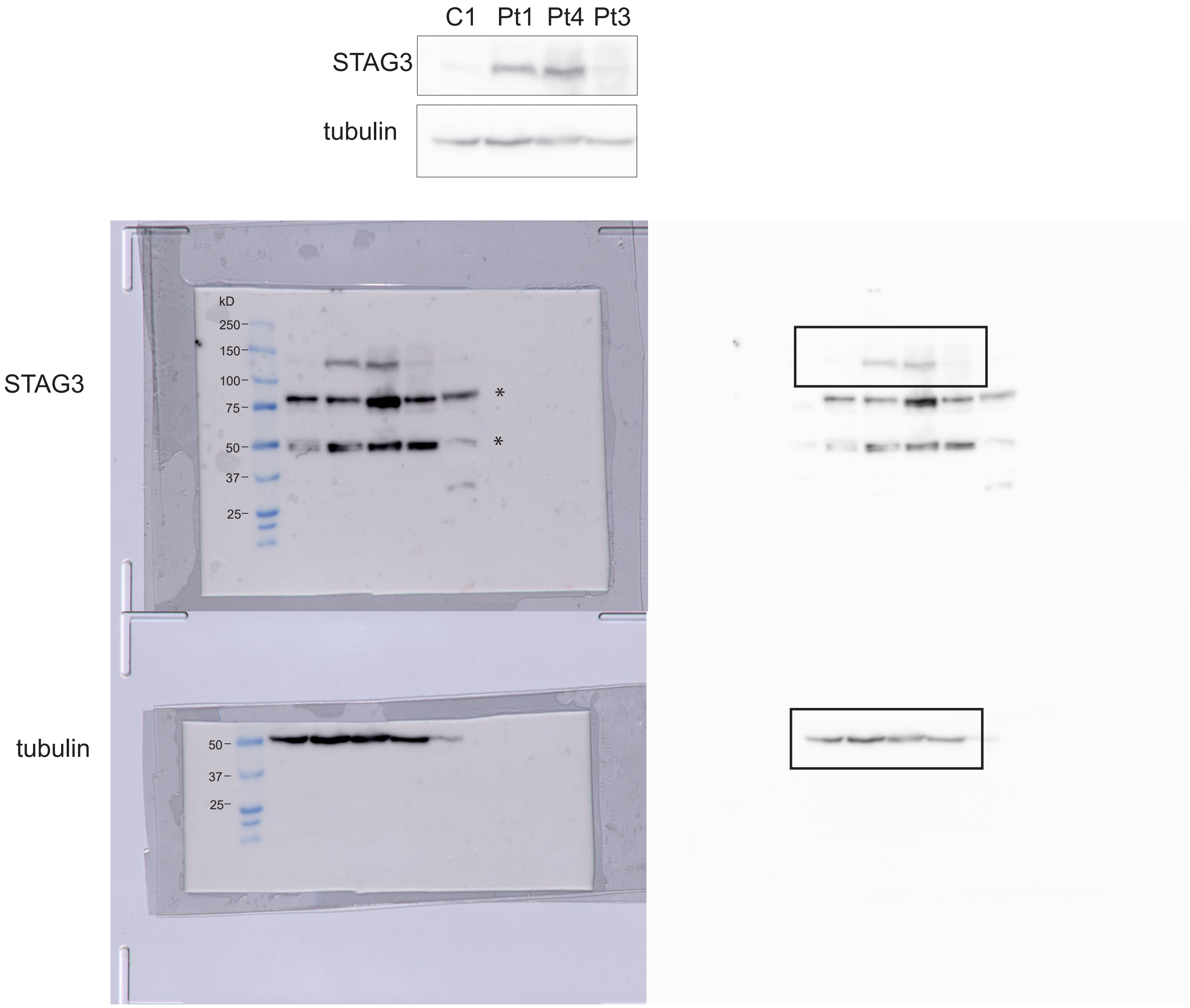

Fig 4B
The left side shows a long exposure of the membrane overlayed with the marker.
A shorter exposure was used to assemble the figure (right side). THe part used for the figure are indicated with boxes.
Note that the membrane shown in Fig 1G was reprobed with rabbit-anti-STAG3 after quenching of the signal. The same for tubulin but here only the lower part of the blot was reprobed.
Note that the STAG3 antibody used shows unspecific bands marked with a star (*).

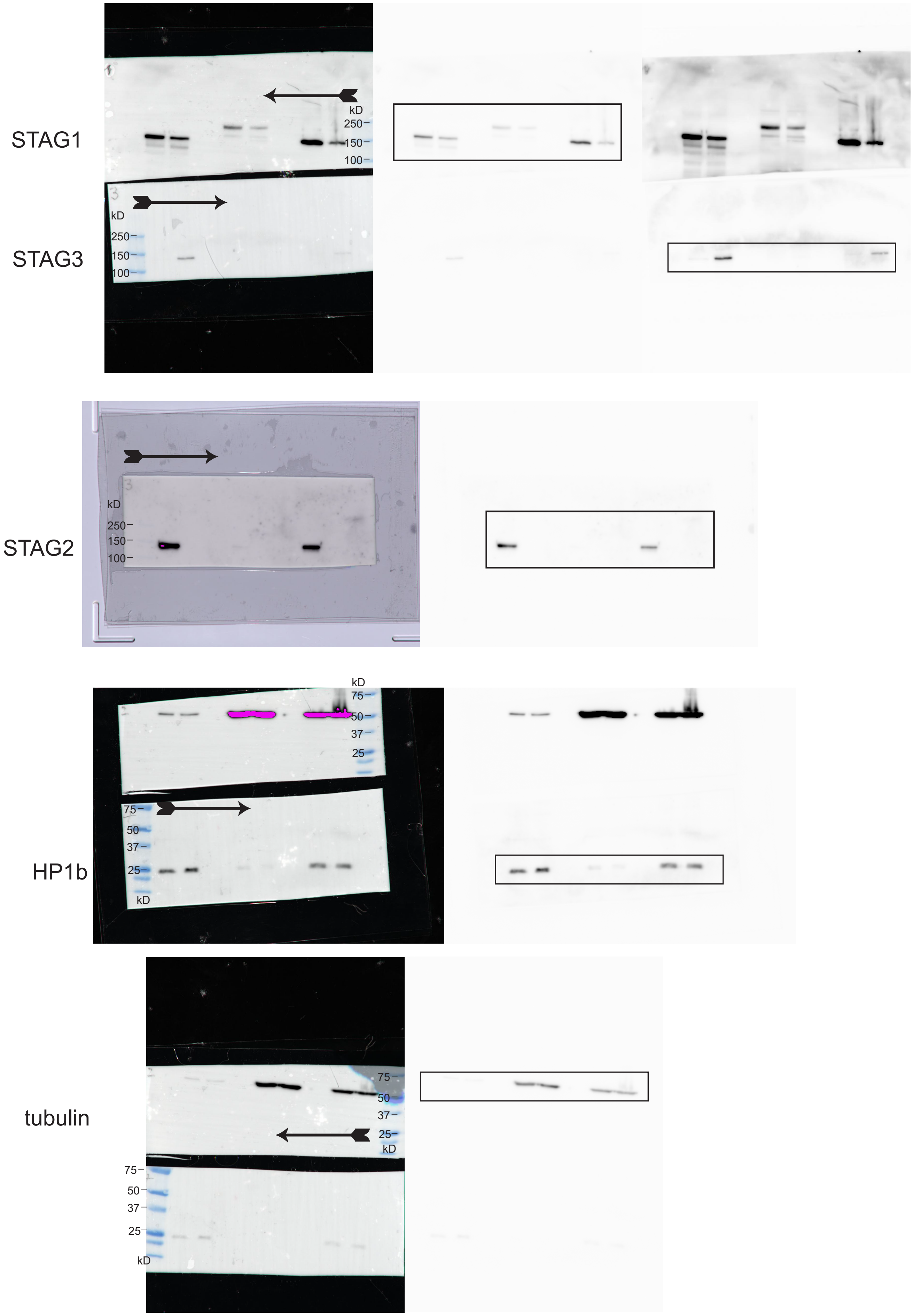

Fig 4D
The left side shows a long exposure of the membrane overlayed with the marker.
A shorter exposure was used to assemble the figure (right side). The parts used for the figure are indicated with boxes. Note that two blots with identical loading were used.
One membrade was probed with anti-STAG1 and the second one succesively with anti-STAG3 and anti-STAG2. The lover part of the membranes were probed with anti-tubulin and anti-HB1b respectively. The direction of loading is indicated with arrows.

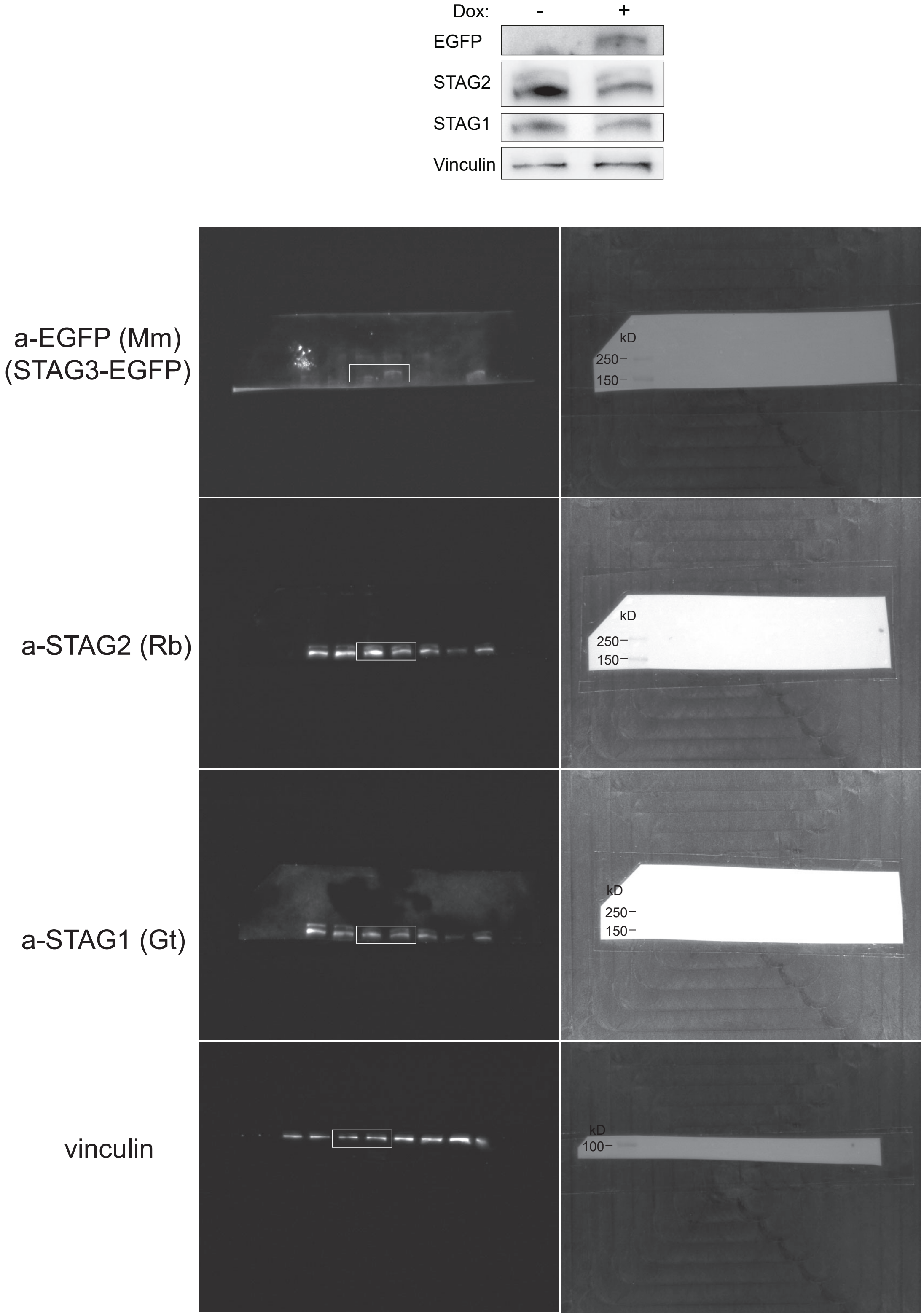

Fig 5A
The top figure shows the figure in the manuscriot. Below, the raw images (left) and the blot image showing the marker are displayed. The bands relevant for the figure are indicated with boxes.
The signlas have been inverted for the figure tomake the signals better visible.
Note that the upper part of the blot was sequentially probed with antibodies against EGFP, STAG2 and STAG1 after quenching of the signal.
The lover part of the membrane was probed with anti-vinculin as loading control.

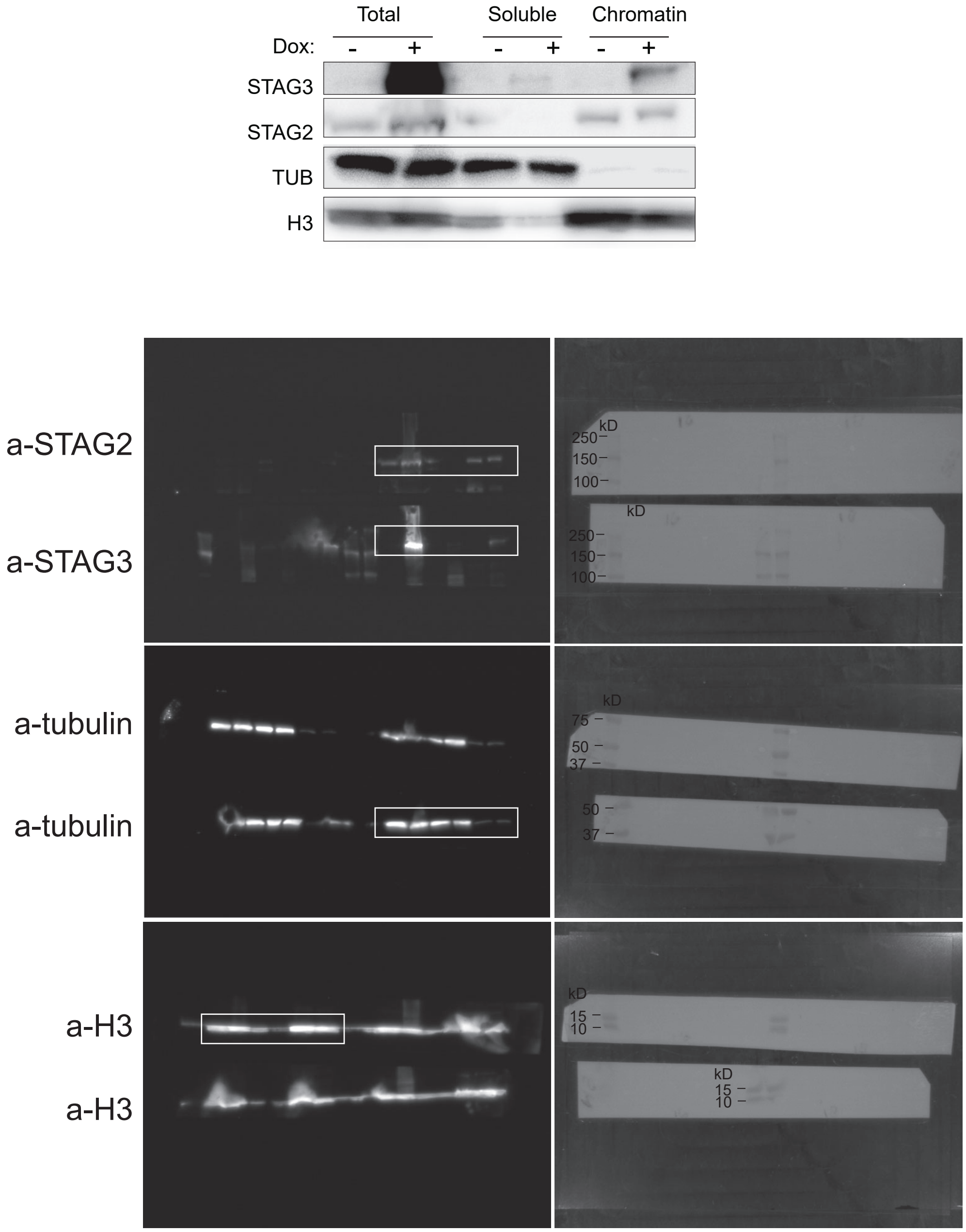

Fig 5B
The top figure shows the figure in the manuscript. Below, the raw images (left) and the blot image showing the marker are displayed. The bands relevant for the figure are indicated with boxes.
The signals have been inverted for the figure tomake the signals better visible.
Note that the same samples were loaded several times to be able to probe with an anti-STAG2 (Rb) and an anti-STAG3 (Rb).
To not overload the blot for blotting Histone H3, at this part of the blot only 20% of the samples used for the other parts was used.

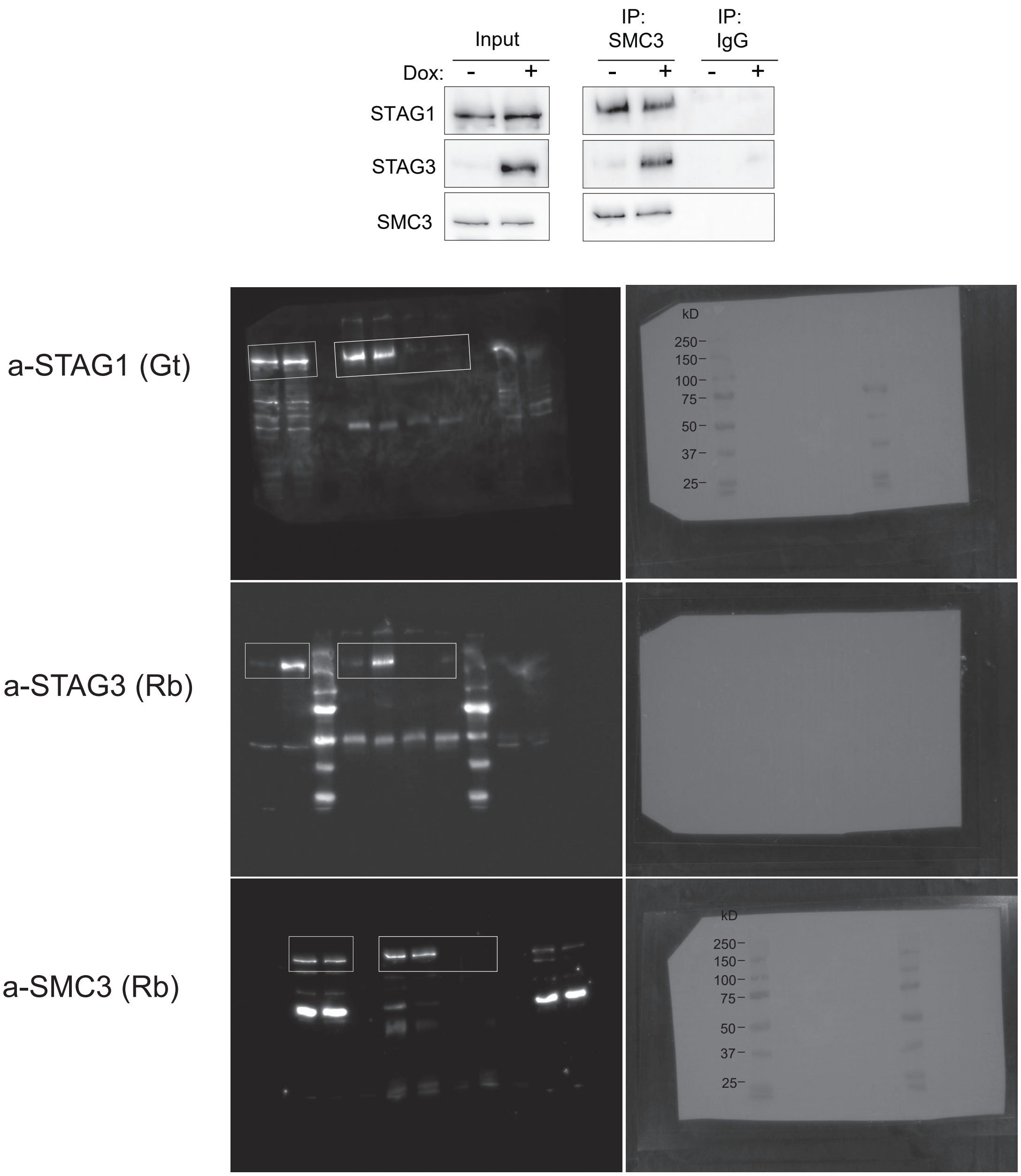

Fig 5C

The top figure shows the figure in the manuscript. Below the raw images (left) and the blot image showing the marker are displayed. The bands relevant for the figure are indicated with boxes. The signals have been inverted for the figure to make the signals better visible.

The immunoprecipitation eluate was split in two and two identical western blots were prepared to allow probing with different antibodies.

Note that the upper part of the blot was sequentially probed with antibodies against STAG1 and STAG3 after quenching. Note that this procesure somtimes ffect the visibility of the marker.

One batch of the STAG3 antibody detected the molecular weight marker. However, we are sure that the boxed signals are specific for STAG3 based on our validation experiments.

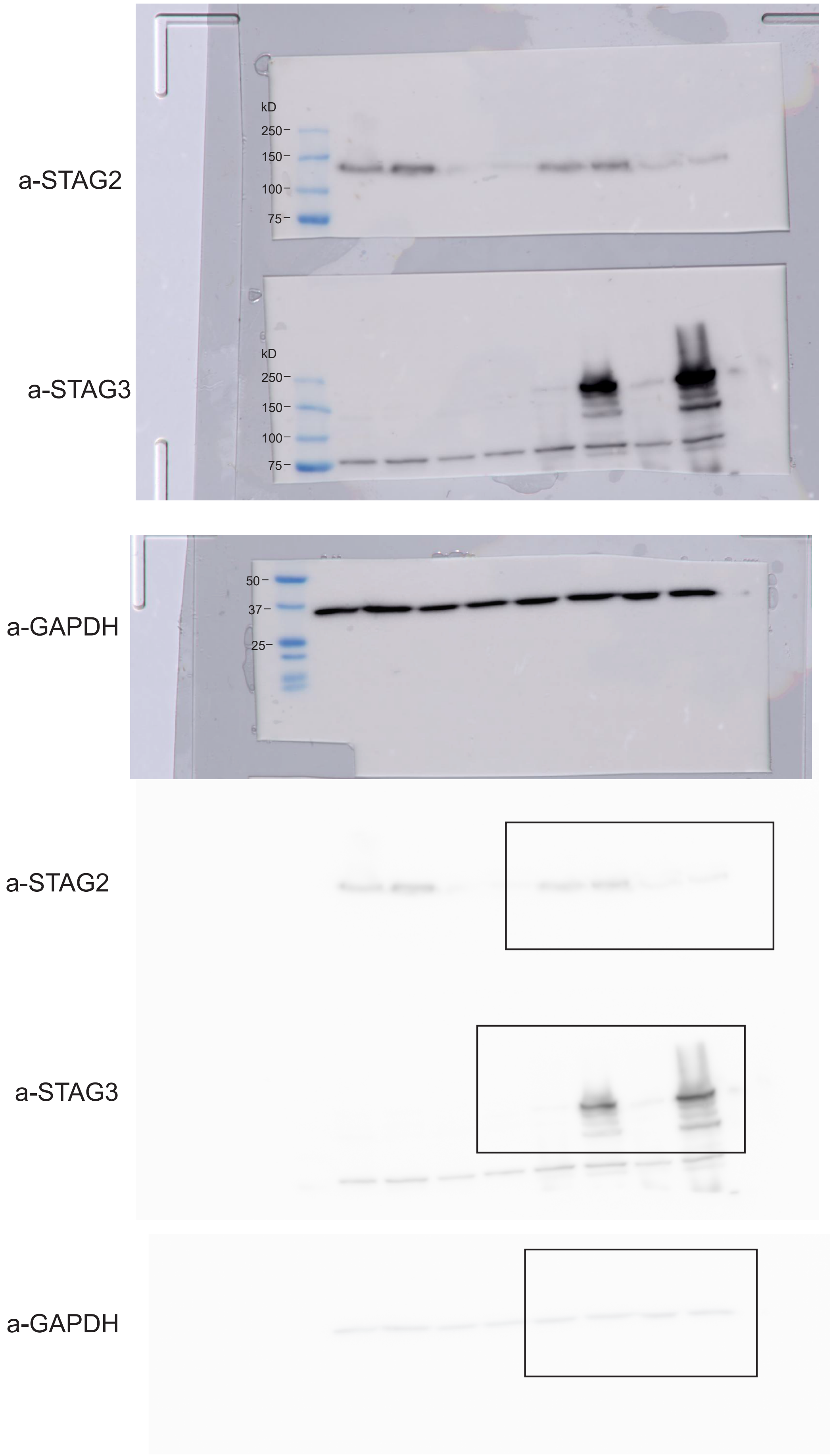

Figure 5D
Upper part: long exposure of the membrane shown together with the molecular marker
Lower part: short exposure, boxes indicate the parts used for the figure

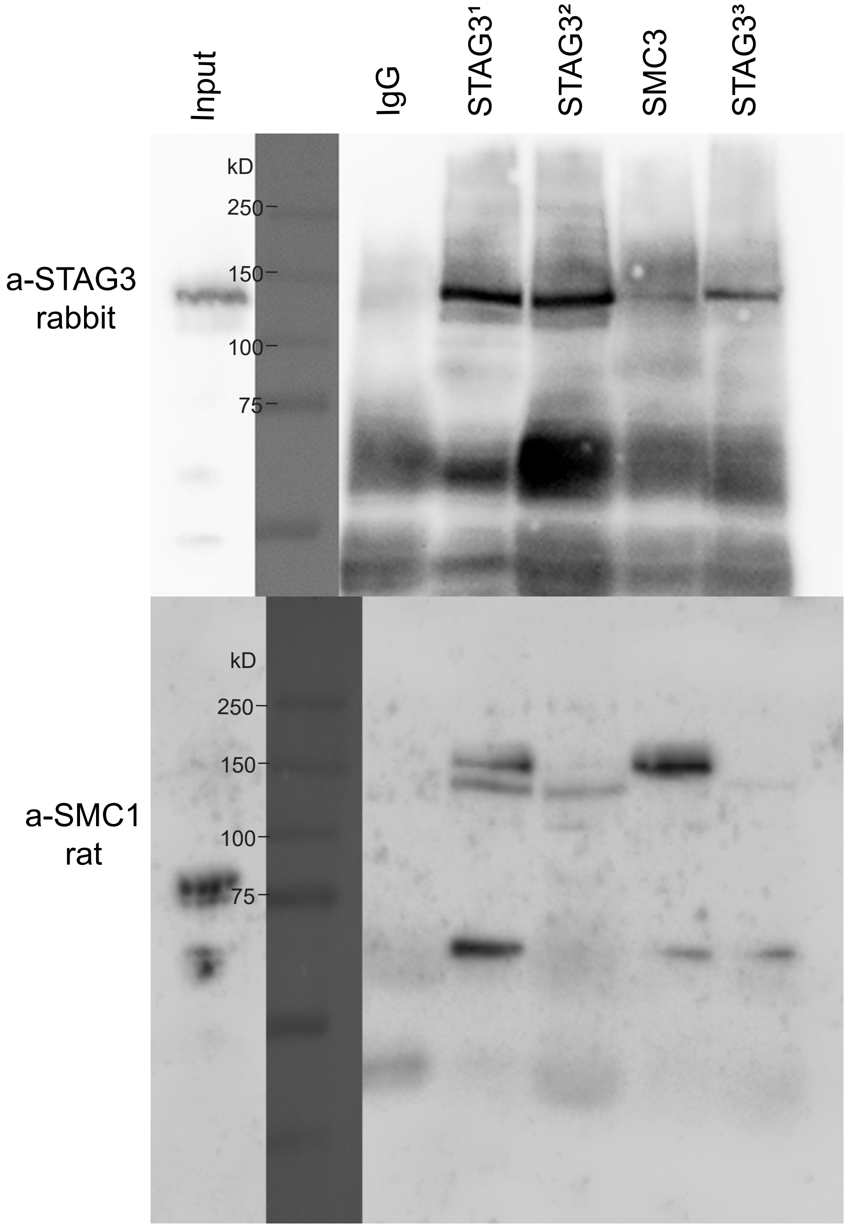

Figure S7B, overlay of uncropped images with the molecular weight marker.
The blot was probed first with anti-STAG3 and after quenching with anti-SMC1A.
